# Let Complexity Bring Clarity: A Multidimensional Assessment of Cognitive Load Using Physiological Measures

**DOI:** 10.3389/fnrgo.2022.787295

**Published:** 2022-02-08

**Authors:** Emma J. Nilsson, Jonas Bärgman, Mikael Ljung Aust, Gerald Matthews, Bo Svanberg

**Affiliations:** ^1^Volvo Cars Safety Centre, Volvo Car Corporation, Gothenburg, Sweden; ^2^Department of Mechanics and Maritime Sciences, Chalmers University of Technology, Gothenburg, Sweden; ^3^Department of Psychology, George Mason University, Fairfax, VA, United States

**Keywords:** physiological measures, cognitive load, driver distraction, psychophysiology, construct validity, measurability

## Abstract

The effects of cognitive load on driver behavior and traffic safety are unclear and in need of further investigation. Reliable measures of cognitive load for use in research and, subsequently, in the development and implementation of driver monitoring systems are therefore sought. Physiological measures are of interest since they can provide continuous recordings of driver state. Currently, however, a few issues related to their use in this context are not usually taken into consideration, despite being well-known. First, cognitive load is a multidimensional construct consisting of many mental responses (cognitive load components) to added task demand. Yet, researchers treat it as unidimensional. Second, cognitive load does not occur in isolation; rather, it is part of a complex response to task demands in a specific operational setting. Third, physiological measures typically correlate with more than one mental state, limiting the inferences that can be made from them individually. We suggest that acknowledging these issues and studying multiple mental responses using multiple physiological measures and independent variables will lead to greatly improved measurability of cognitive load. To demonstrate the potential of this approach, we used data from a driving simulator study in which a number of physiological measures (heart rate, heart rate variability, breathing rate, skin conductance, pupil diameter, eye blink rate, eye blink duration, EEG alpha power, and EEG theta power) were analyzed. Participants performed a cognitively loading n-back task at two levels of difficulty while driving through three different traffic scenarios, each repeated four times. Cognitive load components and other coinciding mental responses were assessed by considering response patterns of multiple physiological measures in relation to multiple independent variables. With this approach, the construct validity of cognitive load is improved, which is important for interpreting results accurately. Also, the use of multiple measures and independent variables makes the measurements (when analyzed jointly) more diagnostic—that is, better able to distinguish between different cognitive load components. This in turn improves the overall external validity. With more detailed, diagnostic, and valid measures of cognitive load, the effects of cognitive load on traffic safety can be better understood, and hence possibly mitigated.

## Introduction

There are many driver states that can affect driving performance, and their contribution to risk increase will remain a central traffic safety study topic until all vehicles are fully automated. For some of these states, such as visual distraction (eyes off road) and drowsiness, the increase in risk is well-documented (Horne and Reyner, 1999; Caird et al., 2014; Victor et al., 2015). For some other states, the contribution to risk increase is less clear. One such state where the effects on driving performance are debated and further studies are needed is high cognitive load, when drivers perform non-visual, cognitively demanding activities while driving (Wijayaratna et al., 2019). (Note the difference between *cognitive demand*, which is something that is posed on the driver, and *cognitive load*, which is the resulting mental response.) It is well-established that, during increased cognitive load, response times to repeated stimuli and artificial tasks increase (Engström et al., 2010; Stojmenova and Sodnik, 2018). In addition, processing of visual information decreases (Strayer et al., 2006) and the gaze becomes more concentrated on the road ahead (Reimer et al., 2012). These findings have led to concerns about missed information and increased brake response times in critical situations (Strayer and Fisher, 2016). However, response times in unexpected critical lead-vehicle braking scenarios appear unaffected by cognitive load (Nilsson et al., 2018) and, in fact, a number of naturalistic driving studies have not found increased crash or near-crash risks for drivers talking on the phone (e.g., Fitch et al., 2013; Victor et al., 2015). It thus still remains to be sorted out when and how cognitive load poses a safety risk (see Engström et al., 2017, and Wijayaratna et al., 2019, for recent reviews and theories).

In order to study the safety impact of cognitive load, we need reliable measures that make it possible to conduct research in more naturalistic settings. The level of cognitive load can then be assessed from the measures instead of being strictly controlled by experimental manipulations. Furthermore, if future studies determine that cognitive load does indeed contribute to elevated risk in certain traffic situations, reliable measures will also be needed so that, for example, Advanced Driver Assistance Systems can detect cognitive load during driving and mitigate its effects by adapting accordingly.

A large number of studies have explored the feasibility of using physiological measures to assess cognitive load (Tao et al., 2019). Advantages of physiological measures are that they can provide continuous recordings of driver states without altering or disrupting the driving task. They can thus complement subjective and behavioral measures (which can also be very informative, but are not the focus of this article) to improve driver state assessments, or be used in situations where subjective or behavioral measures are not sensitive or appropriate (Lohani et al., 2019).

In empirical driving studies today, the level of cognitive load is usually varied systematically by having the participants perform cognitively demanding tasks (from here on referred to as cognitive tasks) while driving. It can, for example, be working memory loading tasks (Heine et al., 2017) or mental arithmetic tasks (Faure et al., 2016). The outcome (i.e., the physiological response) is then typically interpreted as reflecting the level of cognitive load. These studies might conclude, for example, that cognitive load increases the heart rate (Mehler et al., 2012) or the pupil diameter (Niezgoda et al., 2015).

This line of research has provided a great deal of knowledge regarding physiological responses to cognitive tasks. Nevertheless, there are a few well-known, yet commonly disregarded, issues that risk leading to incorrect inferences and generalizations if overlooked. In this article, we wish to bring forward these “elephants in the room,” as we believe that the state of knowledge today allows greater consideration to be given to them.

Cognitive load (also often referred to as mental workload) is commonly defined as the amount of cognitive resources used to meet task demands (Engström et al., 2013; but see Van Acker et al., 2018, for a review and concept analysis). Cognitive resources enable cognitive control, which comprises neurocognitive functions resulting in effortful, conscious, and non-automatized actions (Engström et al., 2013). These functions include, for example, attention, working memory, error monitoring, and inhibitory control (Helfrich and Knight, 2016). Further, cognitive control requires cortical arousal (Grueschow et al., 2020), and can be enhanced (or degraded) by emotional responses (Critchley et al., 2013).

The first issue is thus that cognitive load consists of numerous cognitive and emotional responses that enable cognitive control during increased cognitive demand. (Cognitive and emotional responses will hereafter be jointly referred to as mental responses.) This means that cognitive load is a multidimensional construct and can take many different forms (Matthews et al., 2015). Yet, researchers almost always treat it as unidimensional when attempting to measure it.

The second issue is that task-induced cognitive load does not occur in isolation. Rather, it is part of a complex adaptation to task demands within a specific operational setting (Young et al., 2015). This issue can be best explained in two parts. First, factors other than cognitive task demand may also cause cognitive load and other mental responses, or alter the mental responses caused by the task demand (Van Acker et al., 2018). Such factors can be situation- or human-specific. Situation-specific factors characterize the context in which the task occurs and can, for example, depend on the traffic environment complexity (Törnros and Bolling, 2006; Di Flumeri et al., 2018), time pressure (Loeches De La Fuente et al., 2019), and how many times the task has been repeated (Belyusar et al., 2015). Human-specific factors include the driver's personality (Grassmann et al., 2017), experience (Paxion et al., 2014), and current mental state, such as his/her emotional state (Schoofs et al., 2008) and level of fatigue (Tanaka et al., 2009). The cognitive task and the other influencing factors together affect the driver's mental state and, consequently, his/her physiological responses and behaviors (Faure et al., 2016; Dehais et al., 2020). Second, not all mental responses to changes in cognitive demand are cognitive load components (i.e., mental responses included in the cognitive load construct). Unfortunately, it is often difficult to draw a line between those mental responses that contribute to cognitive control—and are thus to be considered cognitive load components—and those that do not. Mental fatigue is an example of a mental response to (prolonged) cognitive demand that is not part of cognitive load. Stress, on the other hand, is an example of a mental response that is difficult to categorize, since it is beneficial for cognitive control up to a certain limit, after which it has the opposite effect (Dehais et al., 2020).

Lastly, the third issue is that all physiological measures (to the best of our knowledge) correlate with multiple mental responses. These one-to-many relationships limit the inferences that can be made from the individual measures (Richter and Slade, 2017). That is, although many physiological measures correlate with cognitive load, they cannot always be considered measures of cognitive load.

Also, correlation analyses show that physiological measures that correlate with cognitive load are mostly independent of each other, implying that different physiological measures reflect different dimensions in the response to altered cognitive demand (Matthews et al., 2015). There is thus not one physiological response to cognitive load; instead, the physiological responses depend on the mental responses occurring in the specific situation at hand. Multiple physiological measures together can therefore provide us with a more comprehensive idea of the multidimensional cognitive load.

In summary, cognitive load is a complex response to cognitive demands, consisting of multiple mental responses that enable cognitive control. In empirical studies where the level of cognitive demand is altered, many different mental responses occur, depending on the cognitive task, the situation, and the individual. The different mental responses (including the different cognitive load components) have different physiological correlates, as evidenced by the lack of correlation between the physiological measures. Cognitive load can thus be neither measured, nor understood, as a unidimensional and isolated construct, and treating it as unidimensional entails a clear risk of making incorrect inferences and generalizations.

We will suggest that acknowledging these three issues is key to improving the measurability of cognitive load. Here we consider three aspects of measurability: construct validity, external validity, and diagnosticity, because of their relevance in regard to the frequently overlooked issues described above. Construct validity refers to how well a measure actually measures what it claims to measure and encompasses both the measures and the theory behind the construct (Strauss and Smith, 2009). As noted by Strauss and Smith (2009), unidimensional measures of multidimensional constructs are empirically and theoretically imprecise if the construct's components can vary independently, as is the case with cognitive load. External validity addresses the extent to which results from a study apply to other settings (Campbell and Stanley, 1963). Since traffic safety is only relevant in real life, while research on cognitive load is most often conducted in artificial environments, understanding the external validity of measures of cognitive load is highly relevant and deserves more attention than it is usually given (Jiménez-Buedo and Russo, 2021). Diagnosticity addresses a measure's ability to differentiate between different dimensions in the construct it measures (O'Donnell and Eggemeier, 1986), in this case the cognitive load components.

When designing experiments to look for physiological measures of cognitive load, one should bear in mind that the measures are typically sensitive to variations in cognitive load only within certain levels and compositions of load (de Waard, 1996), which vary for different measures and contexts. To improve the chances of finding useful measures, it is thus appropriate to look for physiological measures of cognitive load in settings where there is also an interest in understanding, and possibly mitigating, effects of cognitive load.

The cognitive control hypothesis by Engström et al. (2017) offers a plausible explanation as to how cognitive load affects driver behavior and traffic safety. It states that “*[cognitive load] selectively impairs driving subtasks that rely on cognitive control but leaves automatic performance unaffected*” (Engström et al., 2017, p. 736). Effects of cognitive load are thus relevant when exploring situations where cognitive control is required for a safe outcome; that is, in situations where drivers cannot rely solely on automated behaviors, but need to adapt their behavior using cognitive control (Engström et al., 2017). For example, cognitive load has been found to impair drivers' ability to adapt their behavior to traffic signs (Baumann et al., 2008) and downstream traffic events (Muttart et al., 2007).

On the other hand, automatized driver behaviors should not deteriorate under cognitive load, according to the cognitive control hypothesis (Engström et al., 2017). These behaviors are consistently mapped (i.e., a certain stimulus is consistently followed by the same response) and extensively practiced; one example is normal lane-keeping (Engström et al., 2017). However, if lane-keeping is made more difficult, it can be expected to require cognitive control and thus deteriorate under cognitive load (Engström et al., 2017). In line with this theory, Medeiros-Ward et al. (2014) found that when driving is made less predictable by adding wind gusts, cognitive load led to deteriorated lane-keeping. In contrast, He et al. (2014) found that cognitive load improved lane-keeping also during unpredictable wind gusts. These conflicting results call for further investigation.

As previously mentioned, a driver's mental state, including the loading on different cognitive load components, depends not only on the cognitive task but also on situation- and human-specific factors, one of which is duration. Drivers' physiological responses, behaviors, and mental states may be altered both by prolonged periods of high cognitive demand, and by underload during long-lasting tasks posing only a very low level of cognitive demand, such as simple driving (Saxby et al., 2013; Matthews et al., 2019). Desmond and Hancock (2001) named the two conditions active fatigue and passive fatigue, respectively. Although both fatigue conditions can have negative effects on task performance (Saxby et al., 2013), these effects result from different (and yet not well-understood) neurocognitive mechanisms (Berberian et al., 2017; Hu and Lodewijks, 2020) and thus require different countermeasures (Dehais et al., 2020).

Another situational factor is repetition. In experimental driving studies, tasks, and traffic scenarios are typically repeated, to increase the number of data points to improve statistical stability (Engström et al., 2010). But repeating the same cognitive tasks and traffic scenarios can lead to learning effects, which reduce the level of cognitive load and may alter its composition (Borghini et al., 2016).

### Aim and Approach

The aim of this study is to demonstrate and exemplify how the measurability of cognitive load can be improved by studying multiple mental responses, using multiple physiological measures and independent variables. Changes in mental state are to be assessed based on drivers' physiological responses in relation to three independent variables, namely cognitive task demand, repetition, and traffic scenario. Five analysis questions have been defined:

Q1) How does cognitive task demand affect physiological measures?
Q2) How does repetition affect physiological measures?
Q3) Do the effects of repetition differ when the participant is just driving (baseline) compared to when also doing a cognitive task?
Q4) How do different traffic scenarios affect physiological measures?
Q5) Do the effects of traffic scenario differ when the participant is just driving (baseline) compared to when also doing a cognitive task?

Interpretations about the drivers' mental responses are to be made from answers to these questions in light of the three issues described; (1) cognitive load consists of multiple mental responses, (2) cognitive load does not occur in isolation, and (3) physiological measures correlate with multiple mental responses. The interpretations are to be based on state-of-the-art literature on physiological measures, their mental state correlations, and their neurological underpinnings. While some mental responses are clear cognitive load components, others are relevant because they affect the same physiological measures and could possibly affect the responses to the cognitive demand (e.g., Do et al., 2021).

To pursue this aim, a driving simulator study was conducted in which physiological measures were collected while cognitive task demand was manipulated with a working-memory loading n-back task at two levels of difficulty. The simulated drive consisted of a rural road with three traffic scenarios repeated four times each. When driving through these traffic scenarios, the participants were either just driving (baseline condition), or were concurrently performing the n-back task.

The following section is an overview of the physiological measures that were studied, to facilitate a nuanced discussion on the physiological responses observed in this study.

## Theory

Our bodily functions, and thus physiological responses, are controlled by the endocrine (i.e., hormonal) system and the more rapid nervous system (Tortora and Derrickson, 2007). The nervous system is divided into the central nervous system (CNS), consisting of the brain and spinal cord, and the peripheral nervous system (PNS), which connects the CNS to the rest of the body. Within the PNS, the somatic nervous system controls voluntary movements, while the autonomic nervous system (ANS) exerts involuntary, and often unconscious, control over smooth muscles, cardiac muscles, and glands (Tortora and Derrickson, 2007). The ANS is divided into the sympathetic and parasympathetic nervous systems. In general, sympathetic activation supports emergency reactions (the “fight-or-flight” response), while parasympathetic activation supports activities that occur when the body is at rest (“rest-and-digest” activities) (Tortora and Derrickson, 2007). The two systems can be co-active, reciprocally active, or independently active (Billman, 2013), and different parts of them can be activated separately (Benarroch, 2011). Several interconnected areas in the CNS integrate sensory information with emotional and cognitive processing, control the sympathetic and parasympathetic activity to maintain homeostasis, and facilitate cognitive functions and behavioral responses (Benarroch, 2011). Activity in different parts of the CNS and PNS can be observed through a variety of physiological measures.

### EEG Alpha and Theta Power

Electroencephalography (EEG) is the recording of the electrical activity in the brain's outer cortex. With spectral analysis, the amount of activity (power) of different frequencies within the EEG can be studied. Increased power can be caused by repeated cycles of activation or an accumulation of transient activations (Jones, 2016). The power spectrum is typically split into the frequency bands delta (1–3 Hz), theta (4–7 Hz), alpha (8–12 Hz), beta (15–30 Hz), and gamma (30–100 Hz), although the precise frequency ranges differ between studies (Choi and Kim, 2018). The power in a certain frequency band can be studied either as absolute power or as relative power (absolute power divided by total power) (Choi and Kim, 2018). The two bands most clearly associated with cognitive load are theta and alpha.

Increases in theta power over mid-frontal cortex are frequently related to an increase in cognitive load (Cavanagh and Frank, 2014). But theta power increases also during fatigue caused by either prolonged cognitive performance (Clayton et al., 2015; Tran et al., 2020) or sleepiness (Marzano et al., 2007). In addition, surprising events give rise to transient responses within the theta frequencies (Cavanagh and Frank, 2014). The function of theta activity is not known with certainty, but it seems to reflect a need for cognitive control (Cavanagh and Frank, 2014; Cavanagh and Shackman, 2015). The need can be caused by, for example, a cognitively demanding task or a mismatch between the intended and actual level of attention—due to depleted cognitive resources, as in the case of fatigue (Clayton et al., 2015).

During task execution, alpha power generally increases in task-irrelevant sensory areas in the brain and decreases in task-relevant sensory areas (Pfurtscheller et al., 1996). During cognitive tasks, decrements in alpha power can be spread over several scalp areas (Borghini et al., 2014). Alpha power increase during mind wandering (Compton et al., 2019) and mental fatigue (Borghini et al., 2014). Previously, alpha activity was thought to represent an “idling” of the brain (Pfurtscheller et al., 1996), but theories today attribute it more functions (Halgren et al., 2019). For example, Sadaghiani and Kleinschmidt (2016) suggest that spatially widespread alpha activity contributes to tonic (i.e., slow-changing) alertness, while locally suppressed alpha activity contributes to selective attention by increasing activity and information processing in the area. The alpha band actually consists of several sub-bands, with overlapping frequencies, which respond differently to different tasks and activities (Barzegaran et al., 2017; Benwell et al., 2019).

In driving studies, cognitive task execution have led to both increased theta (frontal) and alpha (frontal, as well as more widespread) power (Sonnleitner et al., 2012; Almahasneh et al., 2014; Wang et al., 2018; Zokaei et al., 2020). During increased driving demand, alpha power has been found to decrease (Wascher et al., 2018; Abd Rahman et al., 2020), while results on theta are mixed (Wascher et al., 2018; Abd Rahman et al., 2020; Diaz-Piedra et al., 2020). Alpha and theta power both tend to increase with driver sleepiness (Simon et al., 2011; Perrier et al., 2016), although not always significantly (Ahlström et al., 2021). The effects of driving time on both bands are mixed (Perrier et al., 2016; Wascher et al., 2018; Ahlström et al., 2021). Noteworthy is that the effects of various activities or conditions can differ, depending on whether absolute or relative power measures are used (Wascher et al., 2018).

### Pupil Diameter

The pupil reacts to the amount of light entering the eye by changing its size (Joshi and Gold, 2020). Additionally, cognitive and emotional states modulate the pupil size (Joshi and Gold, 2020). The pupil diameter (PD) is regulated by the sphincter muscle, which is under parasympathetic control and causes pupil constriction, and the weaker dilatory pupillary muscle, which is under sympathetic control and causes pupil dilation (Larsen and Waters, 2018). A brain area highly involved in the control the pupil size is the locus coeruleus (LC) (Joshi and Gold, 2020); the brain's primary source of the arousal-promoting neurotransmitter norepinephrine (NE) (Samuels and Szabadi, 2008). The LC-NE system plays a crucial role in cognitive processes and task performance and its activity is closely reflected by the PD (Aston-Jones and Cohen, 2005).

Pupillary responses can be studied as phasic responses and tonic levels. Phasic responses are rapid transient dilations which occur spontaneously or in response to an external stimulus (or to the lack of an expected stimulus) (Joshi and Gold, 2020). Tonic levels are studied by measuring averaged PDs, during either baseline or task conditions. A small PD indicates low vigilance or sleepiness (Zénon, 2019), while a large PD reflects high arousal (Aston-Jones and Cohen, 2005) or high levels of cognitive activity (Zénon, 2019). During task execution, a large PD indicates greater effort and often correlates with good performance (van der Wel and van Steenbergen, 2018).

Numerous driving simulator studies have shown increased PD during cognitively (Hammel et al., 2002; Niezgoda et al., 2015; Cegovnik et al., 2018; He et al., 2019; Peruzzini et al., 2019) and visually (Benedetto et al., 2011) demanding secondary tasks, psychological stress (Pedrotti et al., 2014), and time pressure (Rendon-Velez et al., 2016), as well as during increased driving demand (Peruzzini et al., 2019; Xie et al., 2020). As task demands increase, the PD typically shows a stepwise increase before it plateaus or decreases again at high load levels when performance can no longer be maintained (van der Wel and van Steenbergen, 2018). The plateau and decrease are likely due to a decrement in motivation and effort (van der Wel and van Steenbergen, 2018).

Few studies have explored the effects of secondary tasks on pupil diameter in real driving. Because the pupil is very sensitive to lighting variations, task effects risk being masked in real-life environments with fluctuating light levels. Nonetheless, Nunes and Recarte (2002) and Recarte and Nunes (2000) found that the PD increased during the execution of cognitive tasks on real roads, except during simple conversation tasks (Nunes and Recarte, 2002). Further, Ahlström et al. (2021) found a decrease in PD with increased distance driven by sleep-deprived drivers at nighttime.

### Eye Blink Rate and Duration

Eye blinks are essential for lubricating the eyes, but characteristics such as their frequency and timing depend on cognitive and emotional factors as well (Cruz et al., 2011). The eye blink rate (EBR) is positively related to the level of the neurotransmitter dopamine in the brain (Eckstein et al., 2017), although the precise relationship is unknown (Jongkees and Colzato, 2016; Sescousse et al., 2018). Dopamine affects several brain functions, including cognitive control, motivation, and learning (Jongkees and Colzato, 2016). Levels of dopamine that are too low or too high, reflected in low or high EBR, typically mean worse performance (Jongkees and Colzato, 2016; Eckstein et al., 2017) due to depressed prefrontal cortex activation (Dehais et al., 2020).

Brain activity studies have suggested that spontaneous eye blinks provide brief moments of attentional disengagement from an external stimulus in favor of internal processing (Nakano et al., 2013). Blinks occur less frequently during visually demanding tasks, probably to reduce the risk of missing relevant information (Recarte et al., 2008). This reduction in frequency has been demonstrated in laboratory studies (Recarte et al., 2008; Cardona et al., 2011) and in driving studies investigating increased driving demand (Wiberg et al., 2015; Faure et al., 2016; Lobjois et al., 2021). In driving studies applying visually demanding secondary tasks, the effect has not reached significance (Liang and Lee, 2010; Benedetto et al., 2011). Unfortunately, because large saccades (quick movements of both eyes) are often accompanied by blinks (Fogarty and Stern, 1989), comparing EBR between different traffic environments or tasks with different glance behaviors can be problematic (Cardona and Quevedo, 2014).

During cognitive tasks, the EBR increases both in laboratory (Recarte et al., 2008; Magliacano et al., 2020) and driving studies (Nunes and Recarte, 2002; Liang and Lee, 2010; Niezgoda et al., 2015; Faure et al., 2016; Cegovnik et al., 2018; He et al., 2019; Chihara et al., 2020). Although these results are highly consistent, EBR differences between levels of cognitive load are typically small and rarely significant.

The effects of increased visual and cognitive demands on eye blink duration (EBD) are less explored and less consistent. Simulator studies have not found significant effects of either traffic complexity (Faure et al., 2016) or cognitively (Faure et al., 2016) or visually (Benedetto et al., 2011) demanding secondary tasks on EBD. However, studies in real traffic have demonstrated shorter EBDs in drivers compared to their passengers (Takeda et al., 2016), as well as during driving in more demanding traffic situations (Wiberg et al., 2015).

During increased driver sleepiness, EBD increase consistently (Ahlström et al., 2018; Cori et al., 2019). The studies are fewer and results ambiguous regarding the effects of driver sleepiness on EBR (Cori et al., 2019). On the contrary, during increased fatigue due to prolonged task execution, the pattern is reversed: studies consistently show increased EBR while the effects on EBD are mixed (Bafna and Hansen, 2021).

### Heart Rate and Heart Rate Variability

The heart is regulated through both the sympathetic and the parasympathetic nervous systems. Sympathetic activity causes the heart to beat faster and stronger, while parasympathetic activity decelerates the heart rate (HR) (Tortora and Derrickson, 2007). The systems can be activated individually or simultaneously and in the same or opposite directions, but parasympathetic activity is faster and stronger than sympathetic activity (Billman, 2013). A healthy heart has a constantly fluctuating HR (Park and Thayer, 2014), measured as heart rate variability (HRV). The respiratory cycle has a major influence on HRV, as inhalations accelerate the heart and exhalations slow it down (Quintana and Heathers, 2014).

Cardiac activity is most often described by HR and HRV. HRV is an umbrella term for different measures of the fluctuations in the time intervals between adjacent heart beats (for an overview, see Shaffer and Ginsberg, 2017). One common HRV measure (used in this study) is the root mean square of successive differences (RMSSD), which is supposed to reflect parasympathetic activity without much respiratory influence (Laborde et al., 2017).

Cognitive tasks lead to increased HR and decreased HRV in laboratory environments (Hughes et al., 2019), as well as in driving studies in simulators (Belyusar et al., 2015; Hidalgo-Muñoz et al., 2019; Tejero and Roca, 2021) and on real roads (Reimer and Mehler, 2011; Mehler et al., 2012). The effects of driving demand on HR and HRV are however varying. For example, simulator studies by Foy and Chapman (2018) and Stuiver et al. (2014) didn't find any effect of varying driving demands on HR, while Wiberg et al. (2015) did find such an effect in real city driving—but the result was less consistent in highway driving. Further, Dussault et al. (2004) found increased HR in pilots during active flight segments compared to in-flight rest segments, but only during actual (not simulated) flights (Dussault et al., 2005). In a driving simulator study by Beggiato et al. (2019), participants' HR typically decreased when approaching traffic scenarios designed to evoke unease. This could be a sign of attentional focusing and preparation for action as HR decelerations are known to occur in aiming sports before an athlete throws a dart or makes a golf putt, for example (Cooke, 2013).

The effects of prolonged task execution and increased mental fatigue on HR and HRV are inconclusive; in fact, both increased (Matuz et al., 2021) and decreased (Li et al., 2002; Mizuno et al., 2011) parasympathetic activity has been suggested. In driving studies, sleepiness due to sleep deprivation causes HR to decrease and HRV to increase on a group level, but individual variation is large (Buendia et al., 2019; Persson et al., 2020; Ahlström et al., 2021).

In general, emotions characterized by passivity, such as sadness, contentment, and suspense, cause a decrease in HR, whereas the opposite is true for emotions characterized by active coping responses, such as anger, embarrassment, and fear (Kreibig, 2010). As an example, HR increases during emotional stress caused by having one's performance judged (Kelsey et al., 2004). The effects of emotions on HRV are less consistent (Kreibig, 2010). Responses to novel stimuli cause a temporary HR deceleration (Bradley, 2009), due to co-activation of the slower sympathetic and faster parasympathetic systems (Silvani et al., 2016).

### Breathing Rate

Breathing, which is under both voluntary and involuntary control (Homma and Masaoka, 2008) both affects, and is affected by, emotions and cognition (Homma and Masaoka, 2008; Del Negro et al., 2018). Roughly every fifth minute, rhythmic breathing is interrupted by a sigh (Del Negro et al., 2018). Sighs open up collapsed alveoli (Del Negro et al., 2018) and reset the breathing rhythm (Vlemincx et al., 2012). Sighs also occur in response to emotions such as grief and happiness (Del Negro et al., 2018), and cause emotional relief (Vlemincx et al., 2013).

The most frequently studied breathing measure in studies of cognitive load is breathing rate (BrR), which consistently increases during cognitive task execution (Grassmann et al., 2016). In single task studies, BrR has also been successful in discriminating between different levels of cognitive load (Backs and Seljos, 1994; Brouwer et al., 2014; Hogervorst et al., 2014; Hidalgo-Muñoz et al., 2019), but this load level sensitivity seems to disappear in driving studies (Mehler et al., 2009; He et al., 2019; Hidalgo-Muñoz et al., 2019). The effects of traffic complexity on BrR appear inconsistent: Wiberg et al. (2015) found BrR to increase during increased traffic complexity in real driving, while Foy and Chapman (2018) found no such effect in a simulator study.

When drivers are sleepy, BrR has been shown to decrease (Kiashari et al., 2020) and become less regular (Rodrígue-Ibáñez et al., 2011). The few studies that have looked at the effects of prolonged execution of cognitive tasks on BrR show inconsistent results (see Grassmann et al., 2016, for a review). BrR also increases during time pressure (Rendon-Velez et al., 2016) and as a result of emotions such as anxiety (Homma and Masaoka, 2008), fear (Stephens et al., 2010), and amusement (Stephens et al., 2010), while it decreases as a result of calm and positive emotions (Balters and Steinert, 2015).

### Skin Conductance

Electrodermal activity is the change in the electrical properties of the skin, typically measured as skin conductance (SC). As sweat ducts fill with sweat, the resistance of the outer layer of the skin decreases and the conductance increases (Dawson et al., 2016). The sweat glands on the palms and soles are densely distributed and primarily respond to emotional arousal in what is known as psychological or emotional sweating (Baker, 2019). The function of emotional sweating is likely to improve grasping as part of the body's preparation to act or flee (Dawson et al., 2016). Sweating is regulated by the sympathetic nervous system alone, making SC a popular measure of general arousal (Posada-Quintero and Chon, 2020).

The SC is most often quantified as tonic changes of skin conductance level (SCL) and phasic sweat bursts called skin conductance responses (SCRs). SCRs occur as part of the orienting response when attention is directed toward a novel, significant stimulus (Bradley, 2009), and also follow deep breaths and body movement (Dawson et al., 2016). In addition, they occur spontaneously approximately one to three times per minute during rest (Dawson et al., 2016).

In principle, the anticipation and performance of practically any task invoke increased SC (in both SCL and SCRs) (Dawson et al., 2016). Cognitive tasks cause increased SC in laboratory settings (Brouwer et al., 2014; Visnovcova et al., 2016) as well as in driving studies in simulators (He et al., 2019) and real cars (Reimer and Mehler, 2011; Mehler et al., 2012, 2016). The effect of performing a task (compared to a baseline condition) is often greater than the differences between task load levels (Reimer and Mehler, 2011; Mehler et al., 2016), and recovery to baseline levels is rather slow (Mehler et al., 2012; Visnovcova et al., 2016). Studies of driving demand demonstrate that increased traffic complexity leads to increased SC in real (Wiberg et al., 2015) and simulated driving (Foy and Chapman, 2018).

Few studies have been conducted on the effects of sleepiness on SC. Although a decrease in SCR frequency (Michael et al., 2012) and SCL (Miró et al., 2002) has been demonstrated due to sleep deprivation, the effects are rather small and inconsistent—and accompanied by stronger circadian oscillations. As for the effects of emotions, SC typically increases in response to those emotions high in arousal (Kreibig, 2010; Gomez et al., 2016). It has been suggested that the increase in SC reflects motor preparation, as many emotions call for action (Kreibig, 2010). This interpretation explains why emotions related to passivity, such as contentment, relief, and sadness, show decreased SC (Kreibig, 2010).

## Method

The study consisted of two similar test series, Test Series 1 and Test Series 2. Differences consisted of the cognitive tasks employed, and in the design of one of the traffic scenarios (see descriptions in Sections Cognitive Task and Driving Scenarios). Otherwise, the test series were the same. Data were collected at the Swedish National Road and Transport Research Institute (VTI) in Linköping, Sweden. The experiment was approved by the regional ethics vetting board (Regionala etikprövningsnämnden) in Linköping.

### Participants

Participants were recruited from a random selection of the vehicle register over people living in the Linköping area. A total of 70 males participated in the study, 36 in Test Series 1 and 34 in Test Series 2. They ranged in age from 35 to 51 years (M = 43, SD = 4), drove between 50 and 1,200 km/week (M = 309, SD = 205), and had held a driver's license for between 8 and 32 years (M = 23, SD = 5). Additional requirements for participating in the study were to: have normal hearing; have a BMI <30; not rate oneself as extreme in extraversion or introversion, stress-sensitivity, and morning- or evening-type; not have bad health or use medication regularly; not have sleep disorders; and be able to abstain from nicotine for 3 h without withdrawal symptoms. The requirements were there to create a fairly homogenous group of participants to reduce the variance in both mental and physiological responses to the experimental manipulations.

All participants were paid 1500 SEK for their participation.

### Equipment

The experiments were carried out in an advanced moving-base driving simulator. The car body consisted of the front part of a SAAB 9-3 with automatic transmission mounted on a cradle which allowed movement with four degrees of freedom. The field of vision was 120°, and three LCD displays were used to simulate rear-view mirrors. A sound system simulated sounds from the tires and engine. The test leader could communicate with the participants through speakers, which were also used for the cognitive tasks.

Electrooculography (EOG), electroencephalography (EEG), electrocardiography (ECG), electromyography (EMG), skin conductance (SC), and respiratory inductance plethysmography (RIP) signals were recorded using a multi-channel amplifier (g.HIamp, g.tec Medical Engineering GmbH, Austria). Thirty-two EEG channels (Fp1, FpZ, Fp2, F7, F3, FZ, F4, F8, FC5, FC1, FC2, FC6, T7, C3, CZ, C4, T8, CP5, CP1, CP2, CP6, P7, P3, PZ, P4, P8, POZ, O1, OZ, O2, A1, A2) and four EOG channels were recorded using active electrodes on a cap (g.tec g.GAMMAcap), referenced to the right earlobe (A2), and with a ground electrode at AFZ. The EEG electrodes were positioned according to the 10–20 system. Two EOG electrodes were placed horizontally outside the outer canthus of each eye, and two were placed vertically across the left eye. The ECG was recorded with electrodes placed on the right collarbone and a lower left rib. The SC was recorded from the distal phalanges at the index and middle fingers at the left hand, and the RIP with an elastic strap placed around the participant's chest, just below the armpits. The EMG was recorded with electrodes placed on the trapezius (shoulder) and masseter (jaw) muscles. The EMG data was collected for the purpose of EEG artifact handling, but because it was not found to be useful for that purpose, EMG was not included in the analysis and will not be described further. All physiological signals were recorded with a sampling rate of 256 Hz. The EEG, EOG, and ECG signals were band-pass filtered between 0.5 and 60 Hz using an 8th order Butterworth filter and notch filtered between 48 and 52 Hz using a 4th order Butterworth filter. The SC and RIP signals were band-pass filtered between 0 and 30 Hz using an 8th order Butterworth filter.

The pupil diameter was measured with a Smart Eye four-camera system in Test Series 1, and with eye tracker glasses from SensoMotoric Instuments (SMI) in Test Series 2.

### Cognitive Task

The cognitive task was an auditory, non-verbal version of the n-back task (see Mehler et al., 2011, for a similar verbal version). It is well-established that n-back tasks cause increased levels of cognitive load (Jaeggi et al., 2010). A number between zero and nine was orally presented to the participants every other second. If the number just presented was the same as the number presented *n* numbers ago, it was considered a target number. All number series were unique, consisting of 30 numbers, with six target numbers. The participants were instructed to press a button mounted on their right index finger against the steering wheel as soon as they detected a target number. In Test Series 1, that task was only presented at the 1-back (*n* = 1) level, while in Test Series 2, the task was presented at both the 1-back and 2-back (*n* = 2) levels. Right before the task began, the participants were informed through the speakers that the task would begin, and which level it would be.

### Driving Scenarios

The simulated driving environment consisted of a two-lane rural road with a speed limit of 80 km/h. There was occasional traffic, both oncoming and overtaking.

**Figure 1:**
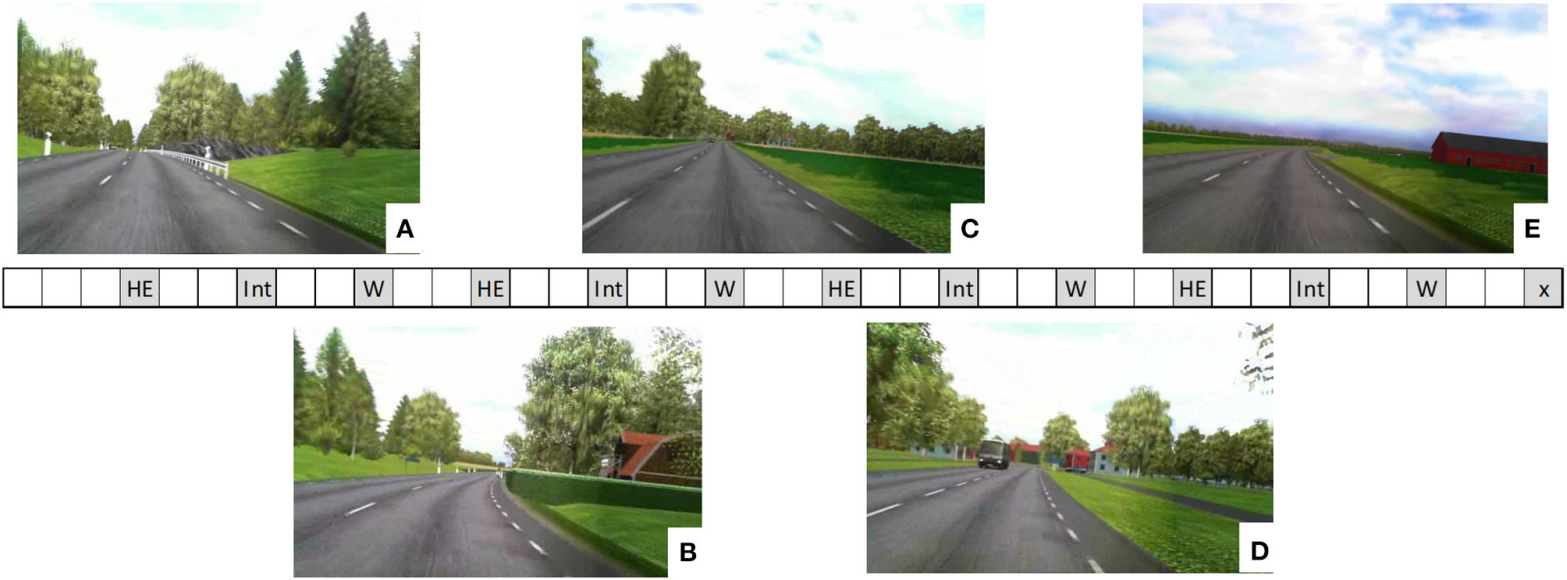
Illustration of the experimental drive. The rectangle in the middle of the figure represents the sequence of events. Each square is ~1 min of driving (depending on vehicle speed). Measurement scenarios are colored in gray. HE, Hidden Exit; Int, Intersection; W, Wind. The final scenario, x, was an unexpected lead vehicle braking scenario which is not included in this study (but see Nilsson et al., 2018). The five images show scenery examples from one of the positions where the hidden exit warning sign became visible for the driver **(A)**; the hidden exit became visible **(B)**; 500 m before the 4-way intersection **(C)**; 80 m before the 4-way intersection **(D)**; and the open field in the Wind scenario **(E)**. Images are blurry due to poor camera resolution, but the participants experienced them in high resolution in the driving simulator.

Measurements were collected during three traffic scenarios (Hidden Exit, Intersection, and Wind), each repeated four times during the drive: see Figure 1. In the Hidden Exit scenario, a warning sign for a hidden exit was placed before a sharp right curve with a high hedge on the right (inner) side. After the curve was the exit on the right side. There was no other traffic in the scenario. In the Intersection scenario, the participants approached and drove through a four-way intersection, in which they had the right of way. Another car approached the intersection from the right, becoming visible as it drove past a house when the participants were 180 m from the intersection, and came to a stop at the intersection 2 s before the participants reached the intersection. A bus in the oncoming lane passed the participant's car 70 m before the intersection. In the Wind scenario, the otherwise present forest surroundings opened up into a field with very limited road curvature. While driving through the field, the participants were occasionally exposed to crosswinds from the right. Wind speed was determined by three overlaid sinusoidal winds with different frequencies, resulting in a, for the participants, unpredictable crosswind.

During the measurement scenarios, the participants were either engaged in the 1-back task or the 2-back task or they were just driving (the baseline condition). In the Hidden Exit and Intersection scenarios, the n-back task started ~45 s before the participants reached the hidden exit or intersection. In the Wind scenario, the crosswinds started 30 s before the task onset and blew for 1 min and 40 s.

The two test series differed somewhat in their design. In Test Series 1, the crosswinds were always active in the Wind scenario. For two repetitions of each scenario the participants were engaged in the 1-back task, and for the other two repetitions, there was no task besides driving (baseline). In Test Series 2, the crosswinds were only active in two of the four Wind scenario repetitions. In each crosswind condition (Wind On and Wind Off), the participants performed the 2-back task once and the baseline condition once. In the Hidden Exit and Intersection scenarios, the participants were engaged in the 1-back task once, the 2-back task twice, and baseline once. The order of the tasks was counterbalanced across participants in both series.

In addition to the measurement scenarios, there were some other scenarios that only differed from the measurement scenarios in terms of traffic, for the sake of variation. There were two more hidden exits with a car standing still at the exit with indicators on, two four-way intersections with a car approaching from the left, and two four-way intersections with no other traffic.

### Procedure

Prior to their participation in the study, participants were sent a background questionnaire and a written description of the study. They were asked to abstain from alcohol for 72 h and nicotine and caffein for 1 h before the experiment. On arrival at the laboratory, they handed in the questionnaire, and the study was once again explained. After the participants gave their written consent to participating in the study, the physiological monitoring equipment was attached. They were then taken to the simulator, the equipment was connected to the measurement devices, and the eye tracker was calibrated.

The participants practiced the 1-back and 2-back (Test Series 2 only) tasks until they felt comfortable performing them. After being informed about the simulator, the participants drove for ~10 min to practice driving. In Test Series 1, the practice drive included two 1-back tasks. In Test Series 2, it included one 1-back and two 2-back tasks. In both series, the practice drive also included one hidden exit scenario with a car standing still at the exit with its indicator on, and one four-way intersection scenario identical to the Intersection scenario.

During the practice session, the participants were allowed to speak to the test leader. Then followed the actual experiment, which lasted ~40 min, during which the participants were asked not to talk to the test leader unless it was urgent. After the drive, participants filled out a questionnaire about their test experience. The overall time, from when participants arrived until they left, was ~3.5 h.

### Physiological Measures

In each measurement scenario, each physiological measure was derived as one averaged value and one continuous vector. The averaged values were computed over 50 s, starting 10 s after task onset. The first 10 s were excluded to reduce the effects of the surprise reaction at task onset. The continuous vectors were initially derived with constant time steps (described for each measure below). The vectors were then transformed to having constant distance steps instead, so that they could be visualized in relation to the traffic environment. The data processing was done in MATLAB R2015b and MATLAB R2019b.

All signals and derived measures in each analyzed segment were visually inspected to ensure adequate data quality before being included in the analysis (a slightly different procedure for the EEG measures is described below). For each measure and scenario, participants were included in the analysis only if they had a complete dataset of all four repetitions.

For EEG data processing, the MATLAB toolbox EEGLAB vs 2020.0 (Delorme and Makeig, 2004) was used. Ninety-second EEG segments, starting 10 s before task onset and ending 20 s after task end (or corresponding segments in the baseline conditions), were extracted. For each segment, all EEG channels were visually inspected. Channels with poor signal quality (either high levels of high frequency noise throughout the recording, or that contained large or frequent signal deviations) were removed. The average number of remaining channels was 27.7 (std 1.5). Also, epochs that contained large movement or muscle artifacts were removed (Tatum, 2014). The remaining channels were then re-referenced to linked ears. To suppress remaining artifacts, which were primarily caused by eye blinks and eye movements, independent component analysis (ICA) was performed on the data, using Infomax ICA (runica, Makeig et al., 1996). The resultant independent components (ICs) were classified into seven categories, including “brain activity” and “eye activity,” using the default classifier in ICLabel (Pion-Tonachini et al., 2019). ICs that were classified as having <20% brain activity or >70% eye activity were removed. The average number of removed ICs was 12.4 (std 3.4). After testing different thresholds on a randomly selected subset of EEG segments, we chose values that led to the exclusion of evident artifacts while retaining as much data as possible. The remaining ICs were subsequently transformed back to the EEG channels. For the averaged values, power spectra were then calculated using Welch's power spectral density estimate with a 2-s window, 50% overlap, and windowing using a Hamming window. The average power was derived for channels F3, FZ, and F4 in the 4–7.5 Hz theta frequency range and for channels P3, PZ, and P4 in the 8–13 Hz alpha frequency range. These averaged power values were then divided by the sum of the total power in the 4–25 Hz frequency range in the same channels, resulting in a relative frontal-midline theta power (Theta) value, and a relative parietal-midline alpha power (Alpha) value. Because artifacts were handled for each segment separately, what ICs were derived and which ones were removed differed between segments. This caused some added variation in absolute power in the processed channels between the segments. Relative, rather than absolute, measures were therefore used as they were less affected by these segment variations. Continuous Theta and Alpha were derived using the same method, but for one 2-s segment at a time, moving in 1/8-s steps, to make continuous vectors. The Alpha vectors were transformed with the natural logarithm to achieve an approximately normal distribution. Alpha and Theta were only derived for the segments in Test Series 2 due to lack of time.

R-peaks were detected in the ECG signals using the *qrsdetect* function in the Biosig toolbox (Vidaurre et al., 2011), and the R-R-intervals (RRIs) were derived as the time between adjacent heart beats. To remove abnormal or artifactual heart beats, RRIs that differed more than 30% from the surrounding six RRIs were removed (Karlsson et al., 2012). The RRIs were then converted to heart rate (HR; beats/min). The continuous HR vectors were derived by linearly interpolating the discrete HR values. Finally, the continuous HR vectors were normalized by subtracting the entire drive's median HR value to reduce between-subject variance in the continuous plots.

The HRV was computed as the root mean square of the successive differences in RRIs after artifact removal (RMSSD) for each 50-s analysis segment (Shaffer and Ginsberg, 2017). The RMSSD values were then log transformed using the natural logarithm to make the distribution more normal (Laborde et al., 2017). No continuous HRV vector was made.

Breaths were detected in the RIP signal using an in-house algorithm based on local peaks and thresholds, and the mean breathing rate (BrR; breaths/min) was derived by counting the number of breaths in the segment. For the continuous BrR vectors, the time between adjacent breaths was derived and converted to breaths per minute. The discrete BrR values were interpolated with next-neighbor interpolation and the vector was normalized by subtracting the entire recording's median BrR value.

In driving studies, SC is commonly studied by averaging of the signal (sometimes after artifact removal or normalization) over relevant segments in time (Mehler et al., 2010; e.g., Loeches De La Fuente et al., 2019). Here, the SC data required some additional signal processing to achieve normal distributions and remove effects of signal drift (which was evident from visual inspection of the signals, as advised by Braithwaite et al., 2015). The following processing steps were conducted. The SC signals were first smoothed with a function, *wsmooth*, based on the Whittaker's smoother (Eilers, 2003), and then filtered using a 2nd order Butterworth lowpass filter with cutoff frequency 0.001 Hz. By subtracting the filtered signal from the SC vector, an SCR vector was derived. The SC vector was then divided by the SCR vector's 99th percentile value (representing that participant's SCR amplitude). The 99th percentile and not the maximum value was used to avoid influence of any rare extreme values or artifacts. This individual response amplitude normalization was necessary to achieve normal distributions. Finally, to compensate for the drift, the vector's average value in the interval 70–10 s before task onset was subtracted from the analysis segment (principle described in Geršak, 2020).

Eye blinks were detected in the vertical EOG signal with an algorithm based on derivatives and thresholds (Jammes et al., 2008). The mean eye blink rate (EBR; blinks/min) was derived by counting the number of eye blinks in the segment. For the continuous EBR vectors, the time between adjacent eye blinks was derived and converted to blinks per minute. The entire recording's median EBR was then subtracted from all EBR data points to reduce interindividual differences. Next, a constant was added to all the data points to make them ≥1, after which they were transformed using the natural logarithm to make them more normally distributed (as suggested by Cruz et al., 2011). Finally, the discrete EBR values were interpolated using next-neighbor interpolation.

Eye blink duration (EBD; ms) was defined as the time between the eye blink's half rise amplitude and half fall amplitude to reduce the problem with otherwise hard-to-define start and end times (as e.g., Ahlström et al., 2018). Eye closures with a duration >500 ms (considered non-blink closures in International Organization of Standardization, 2014) were excluded from the analysis to avoid extreme outliers. The continuous EBD vectors were derived with the same procedure as the continuous EBR vectors.

The pupil diameter (PD; mm) was obtained from the eye trackers. Sudden drops in the PD vector were removed through linear interpolation, to reduce the effects of eye blinks and other tracking issues (Klingner, 2010). In Test Series 2, one PD vector was obtained for each eye and the one with the best signal quality, assessed through visual inspection, was used in the analysis (unless they were both excluded due to poor signal quality). In Test Series 1, only one PD vector was obtained for each subject. The absolute PD values differed between the series, due to the different eye trackers. Both the averaged PD values and the continuous PD vectors were thus normalized by subtracting the entire recording's median PD value. After this normalization, there were no longer any statistically significant differences in absolute PD values between the two series for the same scenarios and task conditions.

### Statistical Analysis

Each physiological measure was analyzed using a mixed model ANOVA with task (baseline, 1-back, and 2-back) and traffic scenario (Hidden Exit, Intersection, and Wind) as categorical fixed-effect variables, scenario repetition (1–4) as a quantitative fixed-effect variable, and test participant as a categorical random-effect variable. Two-way interactions between task and traffic scenario and between task and repetition were included in the model. The significance level was set to 0.05 and Bonferroni correction was used to compensate for the multiple tests. The normality assumption of each ANOVA was confirmed by controlling that its residuals followed an approximately normal distribution (see Supplementary Material; Appendix 1).

In addition, using data from the Wind scenario in Test Series 2, the effects of the crosswinds were tested separately for the task conditions baseline and 2-back (recall that there was no 1-back condition in the Wind scenario in Test Series 2). A mixed model ANOVA was used, with crosswind (Wind on, Wind off) as a categorical fixed-effect variable and test participant as a categorical random-effect variable.

Effects of traffic scenarios were further explored with continuous plots of mean values and their corresponding 95% confidence intervals (CIs), similar to Beggiato et al. (2019). The distributions of the data samples were approximately normally distributed around the means. Note, however, that because the data samples in a continuous plot are not independent from each other, non-overlapping confidence intervals does not necessarily imply a statistically significant difference between two points (Cumming and Finch, 2005). Therefore, paired *t*-tests were made between two points in time (Wind scenario), or position (Hidden Exit and Intersection scenario), for each task condition. The points were chosen so that the level of demand from the traffic scenario was assumed to differ between them, and so that most of the related responses that were visible in the plots took place between them. In the Wind scenario, two tests were made between the point in time where the two greatest wind bursts occurred, compared to 7 s earlier, where the wind was low. In the Hidden Exit scenario, one test was made between the position where the warning sign first became visible to the participant, and the position where the hidden exit first became visible. In the Intersection scenario, one test was made between the position 80 m before the intersection, where the approaching car had slowed down and was approximately one car length from the stopping point, and the position 500 m before the intersection. The position 500 m before the intersection was chosen because it was not clear at what position the participants recognized the scenario, and so a rather large distance to the more demanding part of the scenario was chosen. Examples of what these analysis positions could look like can be seen in Figure 1.

At these points, the average value for each of the physiological measures' continuous vectors in a 2.1 s (Wind scenario), or 42 m (Hidden Exit and Intersection scenario), interval, centered around the analysis point, was derived for each participant and repetition. These averaged values were then used in the paired *t*-tests. Because multiple *t*-tests were made, and the points for testing were selected after the data had been visualized, results need to be interpreted with caution as the risk of type 1 errors is inflated (Forstmeier et al., 2017). Because we want to avoid inflating the risk of type 2 errors and missing actual effects, correction for multiple testing has not been made (Forstmeier et al., 2017). Instead, consistency and effect sizes of visualized and statistically tested effects are considered in the result interpretations.

The mixed model ANOVAs were performed using SAS Enterprise Guide 8.2 and continuous plots and *t*-test with MATLAB R2019b.

## Results

Four of the 70 participants aborted the experiment due to simulator sickness and 3 were excluded from the analysis due to data loss in the logging system. A total of 63 participants were hence included in the analysis. Of these, 9 lacked a complete PD dataset due to logging issues. For one participant in Test Series 2, the n-back task did not start as intended in one Wind scenario, so this participant has three occasions of baseline and only one occasion of 2-back in the four Wind scenarios.

### Crosswind

The mixed model ANOVAs revealed no significant effect of crosswinds in any of the physiological measures, either in the baseline condition or in the 2-back condition: see Table 1. The two crosswind conditions (Wind On and Wind Off) were therefore merged in the remaining analyses.

**Table 1:** Effects of crosswinds on each physiological measure and task condition.

	**Baseline**			**2-back**		
	**Wind On** **m (sd)**	**Wind Off** **m (sd)**	**Main effect**	**Wind On** **m (sd)**	**Wind Off** **m (sd)**	**Main effect**
HR (beats/min)	65.18 (9.34)	65.69 (9.47)	*F*_(1, 30)_ = 1.47, *p* = 0.23	68.79 (9.61)	68.55 (10.01)	*F*_(1, 28)_ = 0.68, *p* = 0.42
RMSSD (-)	3.57 (0.51)	3.53 (0.48)	*F*_(1, 30)_ = 0.40, *p* = 0.53	3.18 (0.48)	3.19 (0.46)	*F*_(1, 28)_ = 0.03, *p* = 0.87
BrR (breaths/min)	15.41 (3.35)	15.60 (3.83)	*F*_(1, 19)_ = 0.15, *p* = 0.70	19.14 (3.45)	19.27 (4.36)	*F*_(1, 17)_ = 0.02, *p* = 0.90
SC (–)	−0.119 (0.143)	−0.011 (0.221)	*F*_(1, 22)_ = 3.81, *p* = 0.06	0.105 (0.314)	0.089 (0.355)	*F*_(1, 20)_ = 0.01, *p* = 0.91
PD (mm)	−0.343 (0.123)	−0.410 (0.171)	*F*_(1, 18)_ = 3.26, *p* = 0.09	0.090 (0.193)	0.036 (0.245)	*F*_(1, 18)_ = 1.84, *p* = 0.19
EBR (blinks/min)	32.66 (13.97)	34.85 (13.02)	*F*_(1, 22)_ = 2.97, *p* = 0.10	36.26 (19.01)	38.19 (19.35)	*F*_(1, 22)_ = 1.44, *p* = 0.24
EBD (ms)	123.9 (23.4)	130.6 (25.2)	*F*_(1, 22)_ = 3.50, *p* = 0.07	123.7 (33.3)	125.4 (35.7)	*F*_(1, 22)_ = 0.15, *p* = 0.70
Alpha (–)	0.0325 (0.0096)	0.0321 (0.0087)	*F*_(1, 23)_ = 0.07, *p* = 0.79	0.0324 (0.0117)	0.0320 (0.0095)	*F*_(1, 21)_ = 0.22, *p* = 0.65
Theta (–)	0.0510 (0.0121)	0.0491 (0.0116)	*F*_(1, 23)_ = 1.30, *p* = 0.26	0.0559 (0.0130)	0.0554 (0.0129)	*F*_(1, 21)_ = 0.07, *p* = 0.79

*No correction has been done to compensate for multiple tests to reduce the risk of type 2 errors. HR, heart rate; RMSSD, root mean square of successive differences between heart beats; BrR, breathing rate; SC, skin conductance; PD, pupil diameter; EBR, eye blink rate; EBD, eye blink duration; Alpha, relative EEG alpha power; Theta, relative EEG theta power; m, mean; sd, standard deviation*.

### Q1) How Does Cognitive Task Demand Affect Physiological Measures?

The mean values, standard deviations, and the number of samples included are presented for each measure in each task condition (all repetitions and scenarios are merged) in Table 2.

**Table 2:** Measure statistics.

	**Baseline**	**1-back**	**2-back**
HR (beats/min)	m = 64.87 sd = 9.37 *n* = 312	m = 67.30 sd = 10.22 *n* = 251	m = 69.04 sd = 10.31 *n* = 177
RMSSD (–)	m = 3.56 sd = 0.48 *n* = 312	m = 3.39 sd = 0.51 *n* = 251	m = 3.20 sd = 0.49 *n* = 177
BrR (breaths/min)	m = 16.96 sd = 3.71 *n* = 219	m = 19.06 sd = 3.06 *n* = 180	m = 19.16 sd = 3.42 *n* = 117
SC (–)	m = −0.030 sd = 0.241 *n* = 270	m = 0.068 sd = 0.249 *n* = 225	m = 0.176 sd = 0.302 *n* = 133
PD (mm)	m = −0.141 sd = 0.246 *n* = 188	m = 0.147 sd = 0.272 *n* = 150	m = 0.334 sd = 0.293 *n* = 114
EBR (blinks/min)	m = 29.21 sd = 12.41 *n* = 244	m = 31.97 sd = 14.27 *n* = 198	m = 35.35 sd = 18.54 *n* = 142
EBD (ms)	m = 122.0 sd = 26.5 *n* = 244	m = 123.9 sd = 32.9 *n* = 198	m = 117.2 sd = 30.0 *n* = 141
Alpha (–)	m = 0.0317 sd = 0.0091 *n* = 92	m = 0.0302 sd = 0.0082 *n* = 45	m = 0.0308 sd = 0.0093 *n* = 135
Theta (–)	m = 0.0515 sd = 0.0121 *n* = 92	m = 0.0549 sd = 0.0117 *n* = 45	m = 0.0556 sd = 0.0121 *n* = 135

*HR, heart rate; RMSSD, root mean square of successive differences between heart beats; BrR, breathing rate; SC, skin conductance; PD, pupil diameter; EBR, eye blink rate; EBD, eye blink duration; Alpha, relative EEG alpha power; Theta, relative EEG theta power; m, mean; sd, standard deviation; n, number of samples*.

Detailed results from the mixed model ANOVAs of the effects of task, repetition, and scenario are presented in Table 3. The task had a significant effect on HR, RMSSD, BrR, SC, PD, and EBR in the form of a stepwise increase (or decrease) with increasing level of cognitive demand in all measures. Only in EBR was the difference between 1-back and 2-back tasks not significant. There was no significant effect of task in EBD, Alpha, or Theta.

**Table 3:** Results from Mixed Model ANOVAs of effects of task, repetition, and scenario for each measure.

	**Main effect task**	***Post-hoc*** **test: Bonferroni corrected** ***p***			**Main effect repetition**	**Solution: estimate (se)**, ***p***			Interaction effect repetition*task	**Main effect scenario**	***Post-hoc*** **test: Bonferroni corrected** ***p***			Interaction effect task*scenario
		**Baseline vs. 1-back**	**Baseline vs. 2-back**	**1-back vs. 2-back**		Repetition*baseline	Repetition*1-back	Repetition*2-back			**Hidden exit vs. Intersection**	**Intersection vs. Wind**	**Hidden exit vs. Wind**	
HR (beats/min)	*F*_(2, 666)_ = 51.48, *p* < 0.0001	<0.0001	<0.0001	0.0002	*F*_(1, 666)_ = 36.84, *p* < 0.0001	0.06 (0.15), *p* = 0.71	−0.97 (0.17), *p* < 0.0001	−0.92 (0.20), *p* < 0.0001	*F*_(2, 666)_ = 12.26, *p* < 0.0001	*F*_(2, 666)_ = 1.57, *p* = 0.21	0.34	1.0	0.41	*F*_(4, 666)_ = 1.69, *p* = 0.15
RMSSD (–)	*F*_(2, 666)_ = 20.60, *p* < 0.0001	<0.0001	<0.0001	<0.0001	*F*_(1, 666)_ = 4.63, *p* = 0.03	0.0184 (0.0109), *p* = 0.09	0.0124 (0.0121), *p* = 0.31	0.0155 (0.0144), *p* = 0.28	*F*_(2, 666)_ = 0.07, *p* = 0.94	*F*_(2, 666)_ = 0.08, *p* = 0.92	1.0	1.0	1.0	*F*_(4, 666)_ = 1.33, *p* = 0.26
BrR (breaths/min)	*F*_(2, 454)_ = 13.74, *p* < 0.0001	<0.0001	<0.0001	0.0001	*F*_(1, 454)_ = 37.45, *p* < 0.0001	−0.56 (0.11), *p* < 0.0001	−0.39 (0.12), *p* = 0.002	−0.42 (0.15), *p* = 0.006	*F*_(2, 454)_ = 0.56, *p* = 0.57	*F*_(2, 454)_ = 2.56, *p* = 0.08	0.78	0.81	0.07	*F*_(4, 454)_ = 0.57, *p* = 0.69
SC (–)	*F*_(2, 564)_ = 13.46, *p* < 0.0001	<0.0001	<0.0001	0.004	*F*_(1, 564)_ = 0.07, *p* = 0.79	0.026 (0.013), *p* = 0.05	0.012 (0.015), *p* = 0.40	−0.031 (0.019), *p* = 0.10	*F*_(2, 564)_ = 3.07, *p* = 0.05	*F*_(2, 564)_ = 4.07, *p* = 0.02	1.0	0.10	0.02	*F*_(4, 564)_ = 2.57, *p* = 0.04
PD (mm)	*F*_(2, 398)_ = 36.77, *p* < 0.0001	<0.0001	<0.0001	<0.0001	*F*_(1, 398)_ = 124.78, *p* < 0.0001	−0.081 (0.011), *p* < 0.0001	−0.112 (0.012), *p* < 0.0001	−0.046 (0.014), *p* = 0.001	*F*_(2, 398)_ = 6.29, *p* = 0.002	*F*_(2, 398)_ = 238.19, *p* < 0.0001	0.27	<0.0001	<0.0001	*F*_(4, 398)_ = 1.69, *p* = 0.15
EBR (blinks/min)	*F*_(2, 514)_ = 11.61, *p* < 0.0001	<0.0001	<0.0001	0.09	*F*_(1, 514)_ = 12.75, *p* = 0.0004	1.32 (0.36), *p* = 0.0003	0.55 (0.40), *p* = 0.16	0.63 (0.47), *p* = 0.18	*F*_(2, 514)_ = 1.19, *p* = 0.30	*F*_(2, 514)_ = 9.78, *p* < 0.0001	0.13	<0.0001	0.04	*F*_(4, 514)_ = 0.94, *p* = 0.44
EBD (ms)	*F*_(2, 513)_ = 1.42, *p* = 0.24	0.12	0.10	0.002	*F*_(1, 513)_ = 118.13, *p* < 0.0001	4.68 (0.84), *p* < 0.0001	7.68 (0.93), *p* < 0.0001	5.47 (1.09), *p* < 0.0001	*F*_(2, 513)_ = 2.89, *p* = 0.06	*F*_(2, 513)_ = 13.45, *p* < 0.0001	1.0	<0.0001	<0.0001	*F*_(4, 513)_ = 1.27, *p* = 0.28
Alpha (–)	*F*_(2, 239)_ = 0.25, *p* = 0.78		0.09		*F*_(1, 239)_ = 10.45, *p* = 0.001	0.0008 (0.0004), *p* = 0.02	0.0005 (0.0005), *p* = 0.35	0.0008 (0.0003), *p* = 0.004	*F*_(2, 239)_ = 0.19, *p* = 0.82	*F*_(2, 239)_ = 3.16, *p* = 0.04	0.74			*F*_(3, 239)_ = 0.88, *p* = 0.45
Theta (–)	*F*_(2, 239)_ = 0.62, *p* = 0.54		<0.0001		*F*_(1, 239)_ = 6.14, *p* = 0.01	−0.0011 (0.0006), *p* = 0.04	−0.0011 (0.0008), *p* = 0.18	−0.0004 (0.0005), *p* = 0.40	*F*_(2, 239)_ = 0.67, *p* = 0.51	*F*_(2, 239)_ = 1.11, *p* = 0.33	0.70			*F*_(3, 239)_ = 1.57, *p* = 0.20

*p-values are reported before Bonferroni correction for multiple tests. A significance level of 0.05 corresponds to 0.006 after Bonferroni correction. Green cells demark a p <0.006. For consistency, results from post-hoc tests of differences between tasks and scenarios are included even where there is no significant main effect, although these effects are not color-coded. Since Alpha and Theta were only derived in Test Series 2, they lack the condition with the 1-back task in the Wind scenario. Thus, it was not possible to derive post-hoc results for all conditions for them. HR, heart rate; RMSSD, root mean square of successive differences between heart beats; BrR, breathing rate; SC, skin conductance; PD, pupil diameter; EBR, eye blink rate; EBD, eye blink duration; Alpha, relative EEG alpha power, and Theta, relative EEG theta power*.

### Q2) How Does Repetition Affect Physiological Measures?

Repetition had a significant effect on HR, BrR, PD, EBR, EBD, and Alpha, but not on RMSSD, SC, and Theta (see Table 3).

### Q3) Do the Effects of Repetition Differ When the Participant Is Just Driving Compared to When Also Doing a Cognitive Task?

BrR and PD decreased significantly with increasing repetition in all task conditions, and the size of the decrease differed slightly between the task conditions for PD (demonstrated by the significant interaction effect between task and repetition; see Table 3). EBR, EBD, and Alpha showed an increasing trend with repetition in all task conditions, but only in EBD did these effects reach significance level in all task conditions. There were no significant interaction effects between task and repetition in EBR, EBD, or Alpha. For HR, the effect of repetition differed between task conditions. While HR decreased significantly with increasing repetition in the 1-back and 2-back tasks, there was no effect of repetition in the baseline condition.

### Q4) How Do the Different Traffic Scenarios Affect Physiological Measures?

The mixed model ANOVAs revealed a significant effect of scenario on PD, EBR, and EBD (see Table 3). Their values for the Wind scenario consistently differed from those of the Intersection and Hidden Exit scenarios (except for EBR which did not differ significantly between Hidden Exit and Wind), while the latter two scenarios did not differ from each other.

**Figure 2:**
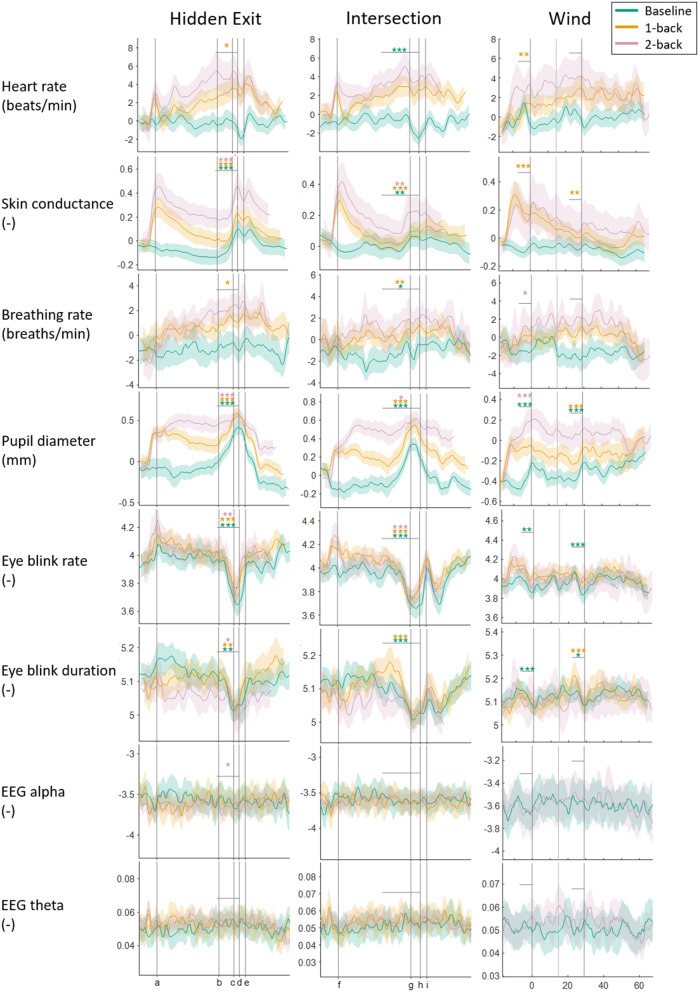
Physiological measures plotted against the specific traffic events for each scenario and task condition. The thick colored lines are the means, and the shaded areas are the 95% confidence intervals. All measures, except Alpha and Theta, are normalized to reduce differences in absolute levels between participants. Curves are slightly smoothed to improve visibility. In the Hidden Exit scenario, the vertical lines show where the task begins (a); the hidden exit warning sign becomes visible (b); the warning sign is (c); the hidden exit becomes visible (d); and the exit is (e). In the Intersection scenario, vertical lines show where the task begins (f); the car approaching the intersection from the right becomes visible (g); the participant's car passes the oncoming bus (h); and the intersection is (i). In the Wind scenario, the three vertical lines mark the peaks of the three strongest crosswinds (the first and third are stronger than the second). Vertical gray lines show between which two points *t*-tests have been done, and the stars above the lines represent the results from the *t*-tests; ***p < 0.001, **p < 0.01, *p < 0.05.

Figure 2 shows the measures' continuous vectors for each scenario and task condition. For the Hidden Exit and Intersection scenarios, the measures are plotted in relation to the traffic environment: the x-axes represent distance driven. The plots are marked where the cognitive task begins and where the participants reach the hidden exit or intersection (depending on the scenario). There is no common position where the tasks end, since that depends on the vehicle speed. The average time between the task onset and the vehicle passing the hidden exit or intersection was ~47 s. In the Wind scenario, the measures are plotted in relation to time, since the wind bursts were controlled by time, not position. Since the tasks' start and end depended on the vehicle speed (both the crosswind and the tasks began at a certain location in the simulated environment), there is neither a common task onset nor end in the plots. On average, the tasks began ~10 s before the first large crosswind (first vertical line).

When the participants approached the hidden exit and intersection, EBR and EBD decreased consistently (except that the EBD decrease in the 2-back condition in the Intersection scenario did not reach significance), while SC and PD increased consistently. Some statistically significant results were found in HR, BrR, and Alpha, but they were inconsistent and small in relation to the signals' overall variability in the segments and are thus less likely to be actual and/or relevant effects.

### Q5) Do the Effects of Traffic Scenario Differ When the Participant Is Just Driving Compared to When Also Doing a Cognitive Task?

The mixed model ANOVAs revealed no significant interaction effects between task and scenario in any measure (see Table 3). However, effect sizes appear to differ between task conditions when approaching and passing the hidden exit and intersection. The increase and decrease in the PD and EBD, respectively, were greater in the baseline and 1-back conditions compared to the 2-back condition. Note that to avoid excessive testing, no statistical testing has been done to compare these effect sizes.

## Discussion

The aim of this simulator study was to demonstrate and exemplify how the measurability of cognitive load can be improved by studying multiple mental responses, using multiple physiological measures and independent variables. We will refer to this as the *multidimensional approach* as it incorporates more than one mental response, measure, and independent variable. With this approach, the three aforementioned issues—(1) cognitive load consists of multiple mental responses, (2) cognitive load does not occur in isolation, and (3) physiological measures respond to multiple mental states—can be taken into account. In this discussion section, the results from the five analysis questions will be interpreted using this multidimensional approach. Some alternative interpretations that overlook the issues will also be provided for the purpose of comparison. This alternative approach will be referred to as a *unidimensional approach*, since it views cognitive load as a unidimensional mental response.

### Effects of Cognitive Tasks

The cognitive tasks had a significant effect on most physiological measures, namely HR, RMSSD, BrR, SC, PD, and EBR, in line with previous research (e.g., Mehler et al., 2009; Faure et al., 2016; Cegovnik et al., 2018). With a unidimensional approach that overlooks the three issues, one could stop the analysis here and conclude that these measures can therefore serve as indicators of cognitive load. We will, off course, not do that.

Note that contrary to what was expected, we saw no effect of the cognitive tasks on the EEG measures Theta and Alpha. One reason could be that we studied relative power instead of the more commonly used absolute power, which (as noted) sometimes show different effects (Wascher et al., 2018). The use of individually adapted frequency bands, instead of fixed bands as was used here, might also improve results (Klimesch, 1999). In addition, the equipment and methods used when deriving alpha and theta power differ a great deal between driving studies, making it difficult to compare results (Choi and Kim, 2018). Thus, the measures' limitations and possibilities in a driving context are still to be determined.

### Including Effects of Repetition

When including the effects of repetition in the mental state assessment through a joint interpretation of HR, PD and RMSSD, the multidimensionality of cognitive load becomes evident. Recall that HR is a frequently used measure of cognitive load (Mehler et al., 2016; Hughes et al., 2019). In line with previous studies, increased cognitive task demand caused a stepwise increase in HR (e.g., Mehler et al., 2010). However, while the HR remained constant in the baseline condition, it decreased with repetition in both task conditions. In other words, the increase in HR caused by the cognitive tasks became smaller over time.

With a unidimensional approach in which cognitive load is viewed as a unidimensional construct whose level is reflected by HR, the decrease in HR would indicate that the level of cognitive load decreased over time. One could then assume, for example, that participants learned the tasks or gradually put less effort into doing them. However, the effects of repetition on PD and RMSSD speak against that interpretation. Increased cognitive task demand caused a stepwise increase in PD and a stepwise decrease in RMSSD, and, importantly, these effects were not attenuated with repetition. (To be precise, there was a significant interaction effect between task and repetition in PD, where the effect of 1-back attenuated slightly and the effect of 2-back increased slightly with increasing repetition. But for the sake of reasonable article length, we will not discuss this further.)

As explained, PD has a close neurological relation to cortical arousal and effort (van der Wel and van Steenbergen, 2018; Joshi and Gold, 2020), whereas the effect of cognitive demand on HR is more complex (Billman, 2013). In studies of mental workload, HR appears more driven by stress and negative emotion than cortical arousal, as the mentioned research on pilots have shown HR to be sensitive to workload alterations in real flying (Dussault et al., 2004) but not in simulated environments, where there is no physical risk involved (Dussault et al., 2005). Furthermore, it has been suggested that HRV has a closer relation to workload than HR (de Waard, 1996).

With the multidimensional approach that acknowledges that cognitive load has multiple components, the combined effects of the cognitive tasks and repetition suggest that different cognitive load components were differently affected by repetition. While the task-induced psychological stress decreased, the increase in cortical arousal and effort remained high throughout the experiment. This indicates that there was no learning or decrease in engagement effects after all.

Effects of repetition are not only seen as changes over time in the mental responses to the cognitive tasks, but also as changes in the participants' baseline state. As the baseline condition was repeated, EBR and EBD increased and BrR and PD decreased. With a unidimensional approach where only the level of cognitive load is of interest, effects of repetition (or time-on-task) are typically rendered insignificant as they are dealt with by employing a randomized or balanced test design. However, employing a multidimensional approach suggests incorporating these effects into the mental state interpretations rather than balancing them out.

The effects of repetition strongly suggest a decline in baseline level of arousal and attention. It seems that the drivers became less engaged with the driving task over time and became more fatigued. Also, HR and RMSSD in the baseline condition remained at relatively low and high levels, respectively, throughout the drive. It thus appears that the level of stress and effort related to the driving task was already relatively low at the first scenario repetition (recall that the participants had first practiced driving before the experimental session began), and that HR and RMSSD are not sensitive to further reductions in driving effort.

The participant's mental state can affect his/her physiological (Conway et al., 2013; Do et al., 2021), behavioral (Schoofs et al., 2008), and mental (Jimmieson et al., 2017; Hidalgo-Muñoz et al., 2018) responses to cognitive demand, so incorporating the baseline mental state when interpreting results improves the external validity and enables better comparisons between studies and environments.

### Including Effects of Traffic Scenario

Here, effects on SC, PD, EBR, and EBD from the cognitive tasks and traffic scenarios are interpreted together. As the drivers approached the intersections and hidden exits, the SC and PD increased and the EBR and EBD decreased. With a unidimensional approach where only the level of cognitive load is assessed, these results appear conflicting. With a multidimensional approach that acknowledges multiple mental responses, these differences are instead informative. Remember that, unlike PD, which increases with increased attention regardless of attention modality, the eye blink measures decrease with increased visual attention, while non-visual attention (such as cognitive tasks) causes an increase in EBR (and sometimes also in EBD) (Recarte et al., 2008). It thus appears that when the participants approached the hidden exits and intersections, their cortical arousal increased due to increased visual attention, together with an increase in general arousal (as reflected in SC; Posada-Quintero and Chon, 2020).

In the case of the wind scenario, any effects of the crosswinds were less pronounced compared with the effects of the environmental demands in the other two scenarios. As noted, previous research employing crosswinds has suggested that the wind poses an additional cognitive demand (Medeiros-Ward et al., 2014), supported by physiological findings: a decrease in Alpha and an increase in Theta (Wascher et al., 2018). In contrast to Wascher et al.'s (2018) findings, there was no effect of crosswind on Alpha or Theta in our study. Recall, though, that there was no effect of the cognitive tasks (which we know cause an increase in cognitive load) on Alpha or Theta in our study, either. These EEG measures do thus not appear sensitive to cognitive load variations in this setting. However, the other physiological measures (which have proven sensitive to variations in several cognitive load components) improve our chances of registering a mental response, if there is one. The mixed model ANOVAs revealed no statistically significant effect of the crosswind on any of the measures, while the effects of individual wind bursts, visualized and statistically tested in Figure 2, showed mixed results. Since no correction for multiple testing has been done on these tests, they should be interpreted with extra consideration of response consistency to avoid type 1 errors. Only PD showed a fairly consistent effect of crosswinds with a significant effect in four out of six tests. It is thus plausible that the participants had brief increases in cortical arousal following the unpredictable crosswinds. But the combined results suggest that the crosswind posed only a very small cognitive load on the participants. Rather, the challenge of driving in a crosswind appears to have been dealt with quite automatically, without the driver having to assert much cognitive control (Schneider and Shiffrin, 1977). Although we employed similar crosswinds to those in Wascher et al.'s (2018) work, our study thus seems to have induced different mental responses. Although the reason is not known at this time, such differences in mental responses between studies could explain observed differences in behaviors between studies (see, e.g,. the different results in He et al., 2014, and Medeiros-Ward et al., 2014).

### Implications of a Multidimensional Approach to Measuring Cognitive Load

The examples above demonstrate how the measurability of cognitive load can be improved by studying multiple mental responses using multiple physiological measures and independent variables. First, acknowledging that cognitive load is a multidimensional construct and measuring (some of) its components individually improves the construct validity of the study, compared to performing a unidimensional analysis (Strauss and Smith, 2009). It is clear from the examples above that several different mental responses occurred during the course of the experiment. For example, the psychological stress that the cognitive tasks gave rise to diminished over time, and visual attention increased with traffic complexity. Until we know how to weight different cognitive load components, it is thus not possible to assess the level of cognitive load on a unidimensional scale.

Having acknowledged that cognitive load is multidimensional and that its components need to be measured individually, the concurrent analysis of multiple physiological measures in relation to multiple independent variables improves the measures' diagnosticity. Making use of the different measures' similarities and differences makes it possible to look at multi-measure response patterns rather than single-measure responses. For example, changes in visual and non-visual attention could be distinguished from each other when PD and EBR or EBD were analyzed together.

At the same time, considering multi-measure response patterns instead of single-measure responses reduces the number of correlations to different mental states. The measurements' context dependence is thus reduced as fewer factors affect the same measurements. This means that the external validity is improved and the risk of making incorrect inferences from observed responses is reduced.

Most research seeking physiological indicators of cognitive load, especially if it employs machine learning, does indeed include multiple measures in the analyses (e.g., Putze et al., 2010; Murphey et al., 2019; Chihara et al., 2020). The use of multiple measures has also been encouraged for a long time (de Waard, 1996). Sometimes, the multiple measures are regarded as “backups” for each other (Tran et al., 2020) to mitigate issues with recording failures (Halverson et al., 2012) or individual response variability (Mehler et al., 2012), but often, multiple measure are indeed combined to improve classification accuracy (i.e., measurability) (e.g., Hogervorst et al., 2014; Prabhakar et al., 2020). However, if cognitive load is not acknowledged as a multidimensional construct, the issue of construct validity and the risk of making inaccurate inferences remain.

One could end up with measures that correlate only with certain cognitive load components that frequently occur in experiments (if that is where the training data are collected)—for example, measures reflecting psychological stress. There is a risk then that these cognitive load components do not occur as frequently in less controlled settings, such as self-initiated cognitive tasks in real-life driving (de Waard, 1996). Consequently, such a measure might fail to detect cognitive load under less stressful circumstances, even if the loading on other cognitive load components is significant.

By measuring and studying cognitive load components separately, researchers can assess the components' individual and combined effects. They can, for example, explore the effects of cognitive effort and psychological stress, separately and together, on driver behavior and traffic safety. Car manufacturers can then use the information gained to prioritize those mental states which are most relevant to detect in Driver Monitoring Systems (DMS), for example. However, there are several challenges when going from group-level studies to continuous monitoring of drivers' mental states.

One great challenge for DMS systems is that between-subject variance in physiological responses to cognitive load (and other mental states) is large (Mehler et al., 2012). Individualized algorithms have therefore been suggested for accurate tracking (Noh et al., 2021). One advantage of tracking multiple mental responses is that the between-subject differences in the physiological responses to changes in individual cognitive load components should be smaller than the differences in physiological responses to cognitive load as a whole (i.e., when it is studied as a unidimensional construct). This is due to the fact that not all drivers have the same mental responses, such as increased psychological stress, during increased cognitive demands (Szalma, 2008). DMS development might thus be somewhat less complicated if cognitive load assessment is made multidimensional.

Still, variability will remain an issue since not all drivers have the same physiological responses to the same mental state changes (e.g., not all individuals display frontal-midline theta activity; Mitchell et al., 2008). While some of this variability could possibly be reduced by breaking down mental responses further, that may render the analysis overly complex. Also, not all cognitive functions can be continuously measured in car drivers. In the end, the appropriate level of detail is one that enables researchers and car manufacturers to understand and, when needed, mitigate any negative effects of cognitive tasks on traffic safety, without making the mental state assessment overly complicated.

It should also be noted that effects seen on a group-level are not necessarily detectable at an individual level because of the multiple factors concurrently influencing the physiological measures. This is especially true where effect sizes are small. For example, the size of mentally driven changes in the PD are typically below 0.5 mm (Beatty, 1982), while alterations in lightening can change the PD several millimeters (Winn et al., 1994).

### Study Limitations

This experiment was designed for many purposes (Nilsson et al., 2018; see also Nilsson et al., 2020), which limited the design possibilities somewhat. Priority was given to achieving a realistic driving task with a low level of interference, which prevented the use of subjective estimates while driving.

The aim of this study was to use multiple physiological measures and independent variables to assess multiple mental responses and, by that, improve the cognitive load measurability. However, only a limited set of physiological measures was included. The measurability can likely be improved using more, and/or other, physiological signals and measures. It may also be that some of the measures are less sensitive in other environments, such as real driving.

The physiological data came from a fairly homogenous group of participants. The variability in responses to the experimental manipulations may therefore be smaller than would have been the case in a more heterogenous group.

Finally, multiple statistical tests were conducted (which is hard to avoid when interpreting multiple measures and independent variables). Bonferroni corrections were made on the ANOVA results to decrease the risk of type 1 errors, while no correction for multiple tests were made for the *t*-test results to avoid inflating the risk of type 2 errors and disregarding actual effects (Forstmeier et al., 2017). To deal with the increased risk of type 1 errors, consistency in results and effect sizes were considered in the interpretations. Still, effects seen in the continuous plots and *t*-tests should be considered exploratory and in need of verification in future studies.

Overall, as the complex relationships between coexisting mental states and physiological responses are still largely unknown, the inferences we made from the physiological measures are, to some extent, speculative. There are also no established “ground truth” measures of mental states to validate our interpretations. Using non-physiological measures, such as questionnaires and performance metrics, could improve the validity of the interpretations (Hancock and Matthews, 2019), although all measures have their own limitations. For example, questionnaires can interfere with the driving task and make it less realistic (O'Donnell and Eggemeier, 1986); people are sometimes not very good at self-assessing their mental state (Schmidt et al., 2009); and performance measures typically have a limited range of sensitivity, since performance can be modulated with effort (Reimer et al., 2012).

## Conclusions

In conclusion, when cognitive load is understood as a multidimensional construct, and (some of) its components are assessed separately using multiple physiological measures studied in relation to multiple independent variables, its measurability can be improved in several ways. For one, the construct validity of cognitive load is improved, which facilitates more accurate and useful result interpretations. Also, studied together and related to multiple mental states, the measures are more diagnostic, in that they are better able to distinguish between changes in different cognitive load components. With multiple measures, multi-measure response patterns can be analyzed instead of single-measure responses. Since the patterns correlate with fewer mental responses, the measurements' external validity is also improved, and the risk of making incorrect inferences from observed responses is reduced.

Improved measurability of cognitive load has the potential to enable more detailed and accurate inferences regarding the effects of cognitive task execution in less controlled settings. As a result, the effects of cognitive load on traffic safety can be better understood and more effectively mitigated.

## Data Availability Statement

The datasets presented in this article are not readily available because they are proprietary. Further inquiries about the dataset can be directed to the corresponding author.

## Ethics Statement

The studies involving human participants were reviewed and approved by Regionala etikprövningsnämnden, Linköping. The patients/participants provided their written informed consent to participate in this study.

## Author Contributions

EN and BS designed the study and contributed to the data acquisition and data curation. EN derived the physiological measures, performed the analyses and visualizations, and wrote the original draft manuscript. EN, JB, ML, and GM performed iterative reviews and edits. JB and ML also supervised EN. All authors contributed to the article and approved the submitted version.

## Funding

The experiments presented herein were part of the Vehicle Driver Monitoring (VDM) project, sponsored by Vinnova, the Swedish governmental agency for innovation systems. The funding agency was not involved in the study design, collection, analysis, interpretation of data, the writing of this article, or the decision to submit it for publication. The final analyses and writing of this article were financed by Volvo Car Corporation. Only the authors analyzed, interpreted the data, and wrote the paper.

## Conflict of Interest

EN, BS, and ML are employed by Volvo Car Corporation. The remaining authors declare that the research was conducted in the absence of any commercial or financial relationships that could be construed as a potential conflict of interest.

## Publisher's Note

All claims expressed in this article are solely those of the authors and do not necessarily represent those of their affiliated organizations, or those of the publisher, the editors and the reviewers. Any product that may be evaluated in this article, or claim that may be made by its manufacturer, is not guaranteed or endorsed by the publisher.
