# Supplementary material for: Let Complexity Bring Clarity: A Multidimensional Assessment of Cognitive Load Using Physiological Measures
Source: Front Neuroergon. 2022 Feb 8;3:787295. doi: 10.3389/fnrgo.2022.787295 (PMC10790847; doi:10.3389/fnrgo.2022.787295)
Supplement: Supplementary file 1 [file Data_Sheet_1.PDF]

Appendix 1. Distribution of residuals

Below are the distributions of the conditional studentized residuals from the mixed model ANOVAs for each physiological measure. Included is a plot of residuals versus predicted values, a histogram with normal density overlaid, a Q-Q plot, and a summary of residual and fit statistics. Analyses and plots are made using SAS Enterprise Guide vs. 8.2.

Heart rate (HR)

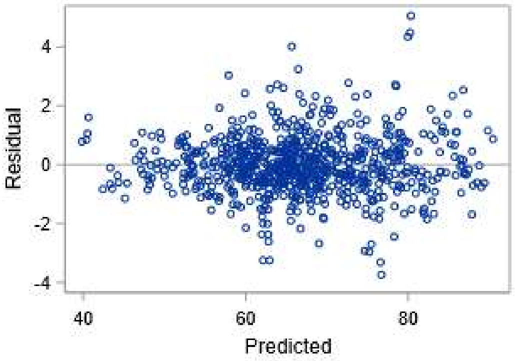

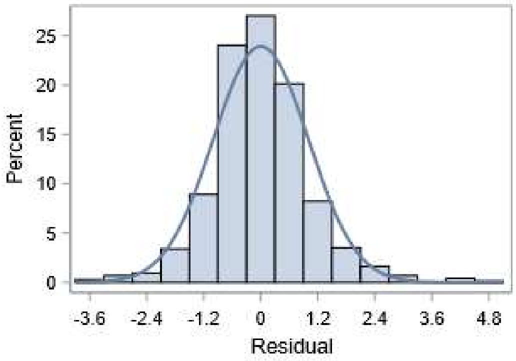

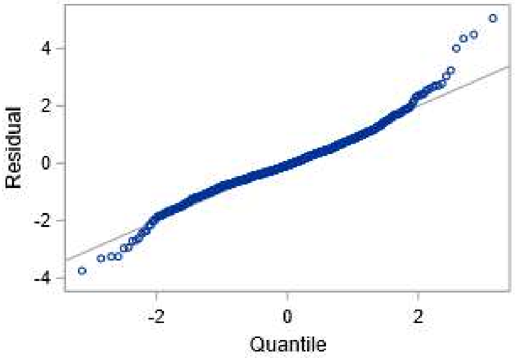

Residual Statistics	
Observations	740
Minimum	-3.741
Mean	191E-7
Maximum	5.0568
Std Dev	1.0001
**Fit Statistics**	
Objective	4006.1
AIC	4012.1
AICC	4012.1
BIC	4018.5

Natural logarithm of RMSSD (RMSSD)

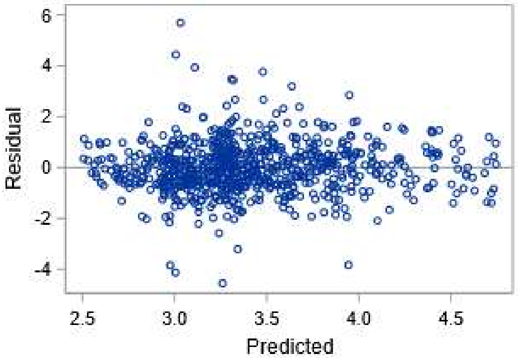

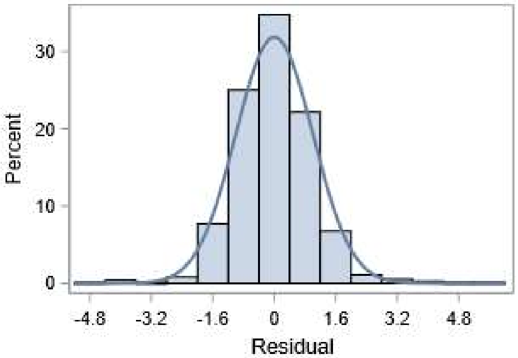

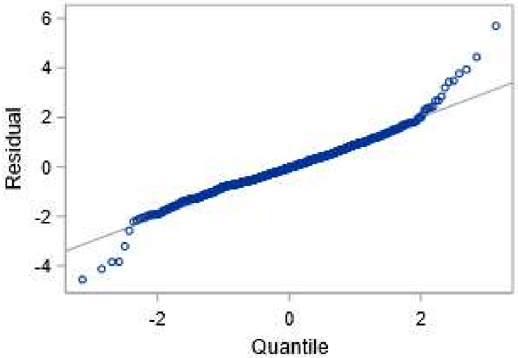

Residual Statistics	
Observations	740
Minimum	-4.551
Mean	-93E-6
Maximum	5.6924
Std Dev	1.0016

Fit Statistics	
Objective	108.57
AIC	114.57
AICC	114.6
BIC	121

Breathing rate (BR)

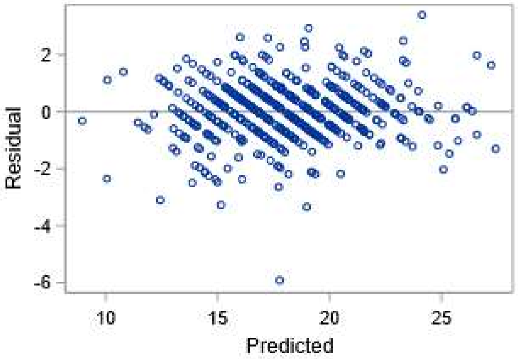

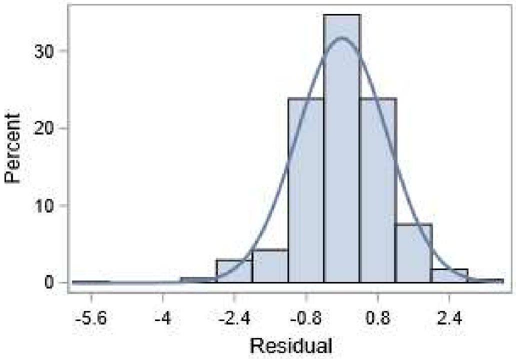

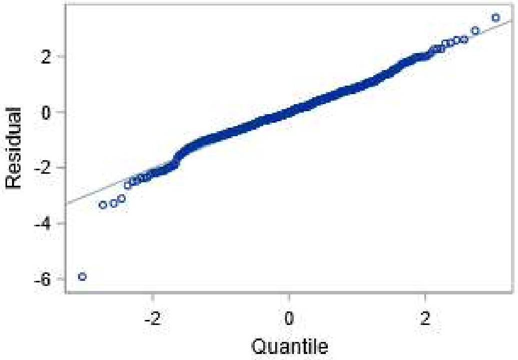

Residual Statistics	
Observations	516
Minimum	-5.914
Mean	0.0001
Maximum	3.3921
Std Dev	1.0078

Fit Statistics	
Objective	2245.5
AIC	2251.5
AICC	2251.6
BIC	2257.3

Normalized skin conductance (SC)

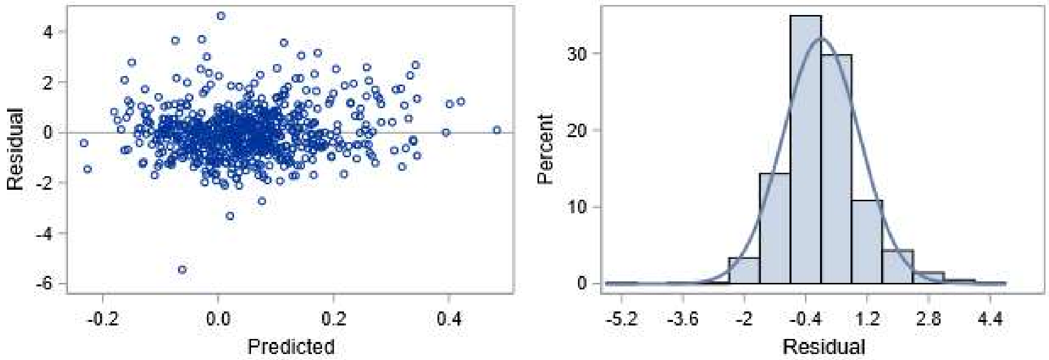

Residual Statistics	
Observations	628
Minimum	-5.439
Mean	-1E-4
Maximum	4.6289
Std Dev	1.0008

Fit Statistics	
Objective	103.38
AIC	109.38
AICC	109.42
BIC	115.29

Pupil diameter (PD)

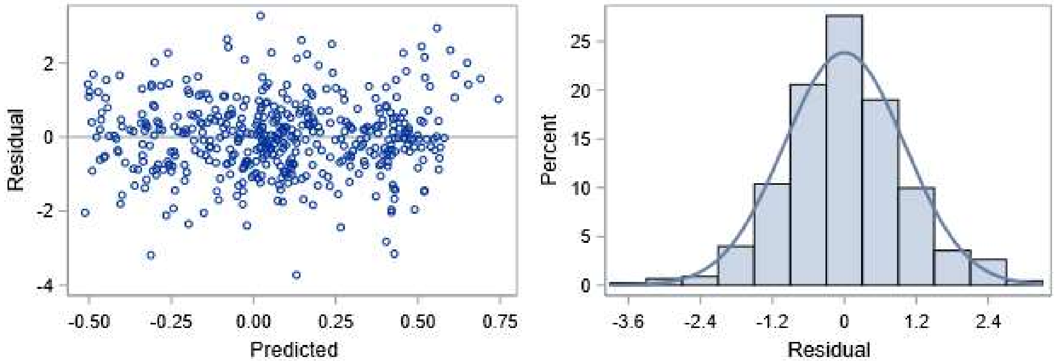

Residual Statistics	
Observations	452
Minimum	-3.727
Mean	0.0003
Maximum	3.2769
Std Dev	1.0033

Fit Statistics	
Objective	-241.9
AIC	-235.9
AICC	-235.9
BIC	-230.6

Eye blink rate (EBR)

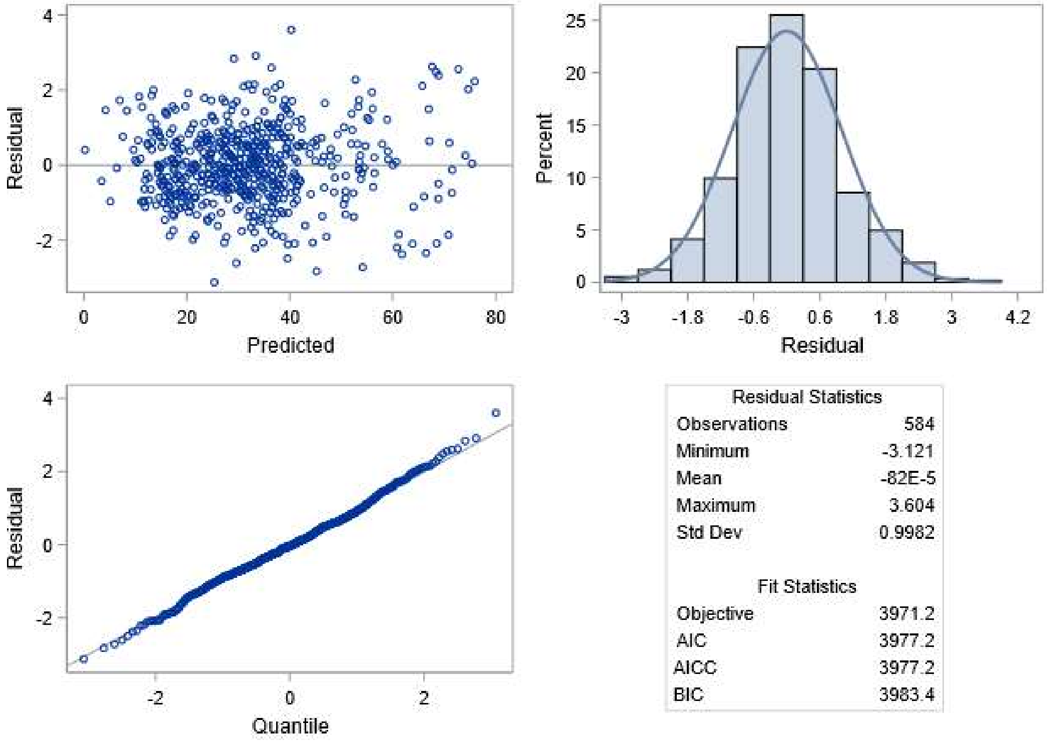

Eye blink duration (EBD)

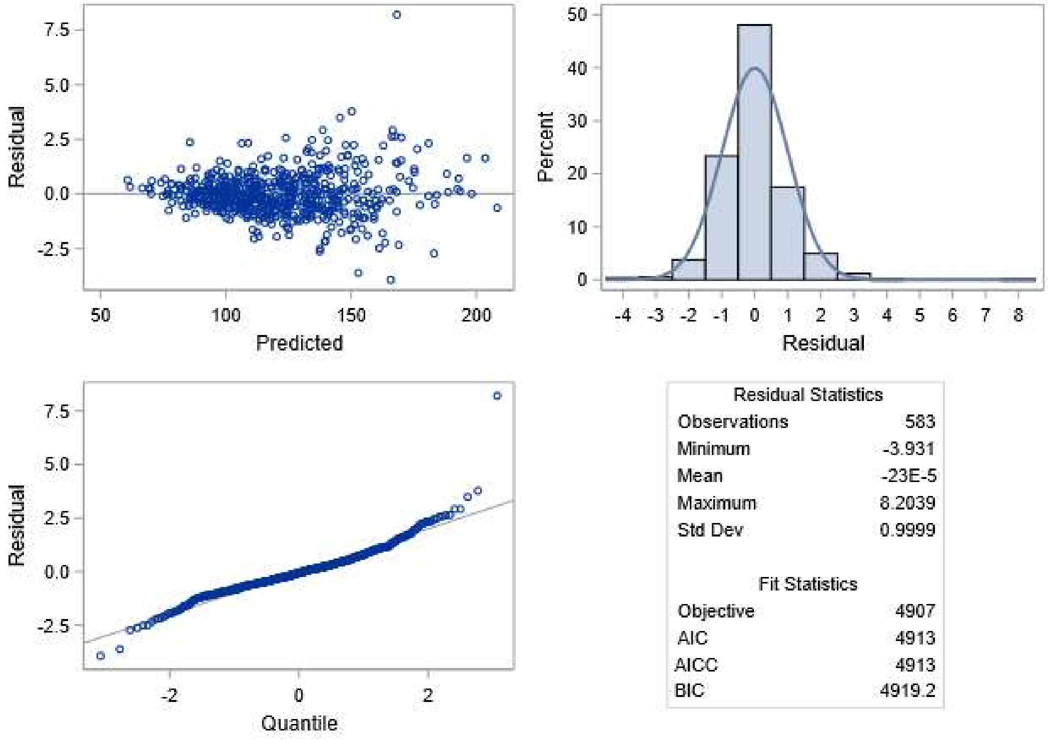

Relative PM alpha power (Alpha)

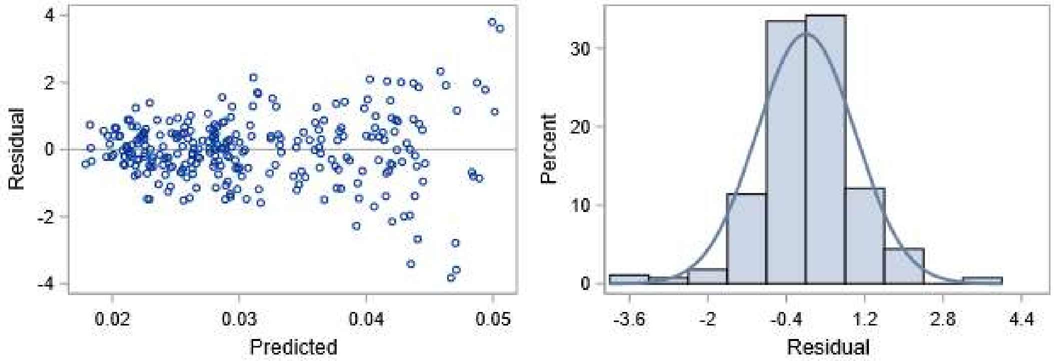

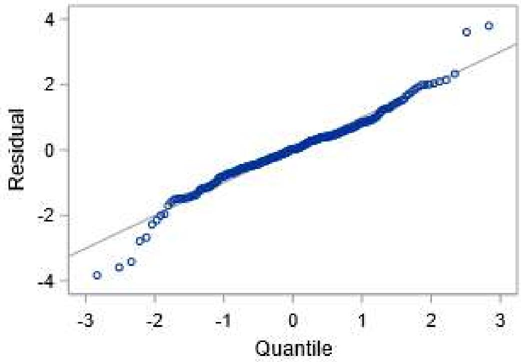

Residual Statistics	
Observations	272
Minimum	-3.827
Mean	-45E-5
Maximum	3.7997
Std Dev	1.0025

Fit Statistics	
Objective	-2063
AIC	-2057
AICC	-2057
BIC	-2054

Relative FM theta power (Theta)

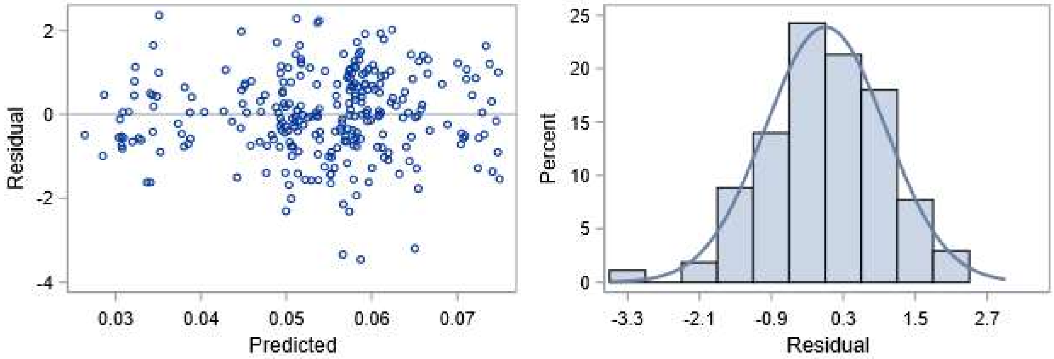

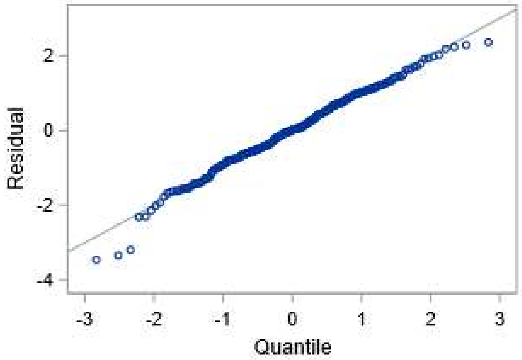

Residual Statistics	
Observations	272
Minimum	-3.461
Mean	0.0004
Maximum	2.3633
Std Dev	1.0011

Fit Statistics	
Objective	-1827
AIC	-1821
AICC	-1821
BIC	-1818
